# Deep Learning Tone-Mapping and Demosaicing for Automotive Vision Systems

**DOI:** 10.3390/s23208507

**Published:** 2023-10-17

**Authors:** Ana Stojkovic, Jan Aelterman, David Van Hamme, Ivana Shopovska, Wilfried Philips

**Affiliations:** IMEC, IPI (Image Processing and Interpretation), Ghent University, 9000 Ghent, Belgium; david.vanhamme@ugent.be (D.V.H.); ivana.shopovska@ugent.be (I.S.); wilfried.philips@ugent.be (W.P.)

**Keywords:** tone-mapping, high dynamic range imaging, deep learning, object detection, systems for automotive driving

## Abstract

High dynamic range (HDR) imaging technology is increasingly being used in automated driving systems (ADS) for improving the safety of traffic participants in scenes with strong differences in illumination. Therefore, a combination of HDR video, that is video with details in all illumination regimes, and (HDR) object perception techniques that can deal with this variety in illumination is highly desirable. Although progress has been made in both HDR imaging solutions and object detection algorithms in the recent years, they have progressed independently of each other. This has led to a situation in which object detection algorithms are typically designed and constantly improved to operate on 8 bit per channel content. This makes these algorithms not ideally suited for use in HDR data processing, which natively encodes to a higher bit-depth (12 bits/16 bits per channel). In this paper, we present and evaluate two novel convolutional neural network (CNN) architectures that intelligently convert high bit depth HDR images into 8-bit images. We attempt to optimize reconstruction quality by focusing on ADS object detection quality. The first research novelty is to jointly perform tone-mapping with demosaicing by additionally successfully suppressing noise and demosaicing artifacts. The first CNN performs tone-mapping with noise suppression on a full-color HDR input, while the second performs joint demosaicing and tone-mapping with noise suppression on a raw HDR input. The focus is to increase the detectability of traffic-related objects in the reconstructed 8-bit content, while ensuring that the realism of the standard dynamic range (SDR) content in diverse conditions is preserved. The second research novelty is that for the first time, to the best of our knowledge, a thorough comparative analysis against the state-of-the-art tone-mapping and demosaicing methods is performed with respect to ADS object detection accuracy on traffic-related content that abounds with diverse challenging (i.e., boundary cases) scenes. The evaluation results show that the two proposed networks have better performance in object detection accuracy and image quality, than both SDR content and content obtained with the state-of-the-art tone-mapping and demosaicing algorithms.

## 1. Introduction

Automotive vision needs to cover a wide range of illumination levels spanning multiple orders of magnitude. Not only is there a large overall difference between daytime and nighttime driving, but even within one scene there can be both deep shadows and blinding (head)lights, making pedestrians and other hazards hard to detect. Automated driving systems need to be able to reliably detect infrastructure and road users to ensure road safety. This implies a necessity for cameras and a video processing pipeline for high dynamic range (HDR) content. The state-of-the-art object detection approaches used in automotive vision rely on standard dynamic range (SDR) 8 bits per color channel neural networks, as in, e.g., YOLOv3 [1]. The consequence is a need to “compress” the dynamic range as captured, a task which is called tone-mapping. Ideally, this happens with as little loss of information as possible. In the processing pipeline, a step further would be to account for optimal reconstruction quality in the diverse challenging traffic scenes by also considering raw HDR data as input.

**Figure 1:**
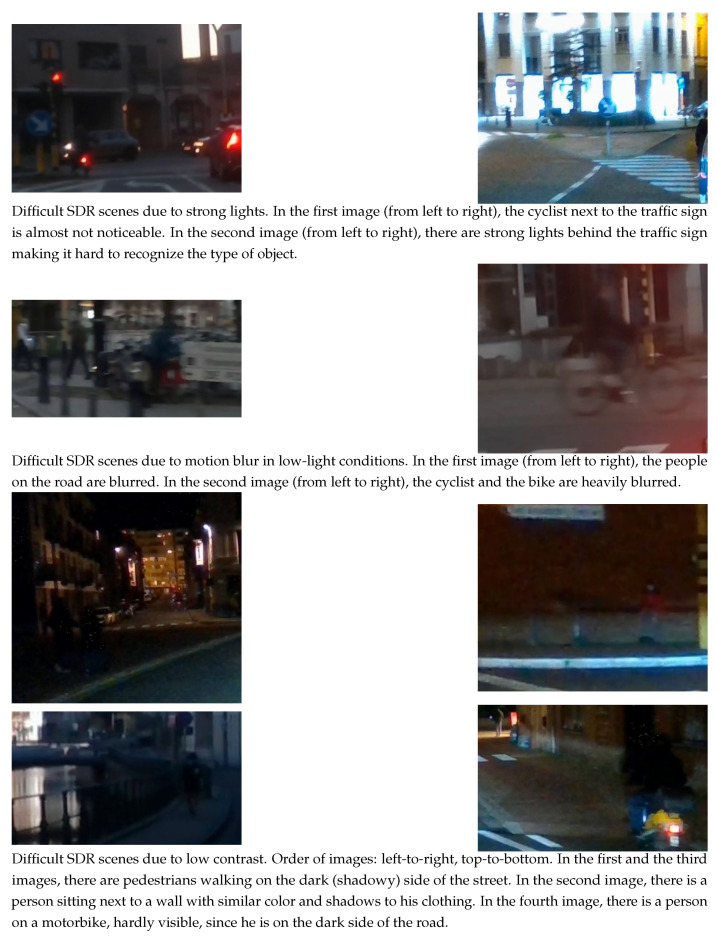
Examples of challenging cases for object detection in SDR content.

Figure 1 shows some examples of challenging traffic scenes for SDR-based object detection. The challenging cases cover motion-blur, the existence of strong lights, low-light conditions, poor contrast, etc.

The goal of this research is to increase the detectability of traffic-related objects in diverse scenes and conditions to improve road safety while using ADS. The three types of approaches that are used currently to address this problem are tone-mapping approaches (that make use of the HDR data), object detection approaches (mainly devised to work on 8-bit content) and reconstruction (demosaicing and denoising) approaches. We focus on tone-mapping and demosaicing.

The rest of the paper is structured as follows. In Section 2, we discuss the related work for the demosaicing and the tone-mapping approaches, both in general and with respect to object detection, by presenting a review of both the state-of-the-art and the most recent algorithms. Then, we present the main novelties of the performed research. Additionally, we briefly describe the architecture of the neural network (devised for inverse-tone-mapping) that was used as starting point architecture for the approach presented in [2], upon which we build our research. In Section 3, we propose two CNN architectures and describe their design and the applied training procedures in more detail. In Section 4, we present the methodology for the performed evaluation analysis, while in Section 5, we present and discuss the results from the quantitative and qualitative analyses. In Section 6, we summarize the conclusions derived from the performed evaluation and discuss the future prospects of the presented research.

## 2. Related Work

Here, we first give a short review on the research studies and algorithms for tone-mapping. Next, we refer to the studies that address the problem of using HDR content for the purposes of object detection. Then, we present a short review of the vast amount of research in the demosaicing field. We then present the novelties of the performed research, by addressing the research gap in which we aim to contribute. Finally, we proceed with a description of the original architecture of the neural network that was used as starting point for the proposed CNNs.

### 2.1. Tone Mapping

Since the arrival of digital photography and video cameras in the 1990s, their resolution and dynamic range capabilities have been steadily increasing.

In general, tone-mapping is defined as the mapping of image tones from one domain to another [3]. However, there is a distinction based on whether the output of the processing is content in the lower dynamic range or the high dynamic range. As mentioned earlier, high dynamic range content can represent a wider range of brightness and colors and hence, is encoded in floating point precision, while standard dynamic range (SDR) content is commonly encoded in 24 bits per pixel (8 bits for every color channel). Some authors [3,4] refer to lower dynamic range content as “display-referred”, since such content is always meant to be displayed, and to HDR content as “scene-referred”, since such content is directly related to the physical properties and the lighting conditions of the captured scene. For this reason, HDR content is often used in applications [5,6,7] where these physical properties are to be measured, such as physically based rendering and image-based lighting, automotive applications, remote sensing, medical imaging, etc. Following a similar analogy, a classification based on the nature of the source data also exists in HDR imaging approaches. Despite the general definition of tone-mapping given above, approaches that perform compression of the dynamic range are commonly referred to as “tone-mapping” approaches, and we refer to them in this way in this work, while approaches that perform conversion from a legacy low dynamic range content into content of a higher dynamic range are referred to as “inverse tone-mapping” approaches. Based on this nomenclature, the devised approaches that are the focus of this research belong to the “tone-mapping” category.

Different authors have compared and categorized HDR approaches according to different criteria [3,4,5,6,7,8,9]. However, generally, algorithms for tone-mapping are classified into global [10,11,12,13,14,15,16,17,18,19] and local methods [20,21,22,23,24,25,26,27,28,29].

In global methods, a tone curve is applied in a pixel-wise manner depending on the measured per pixel luminance of the HDR input. For preserving the contrast, some methods apply linear scaling [10], while others use exponential or logarithmic functions [11,12]. Very often, due to their resemblance to the response curve of the HVS, logistic, i.e., sigmoid, functions have been used for tone-mapping [14,16,18,25]. To achieve better contrast, exposure and detail visibility, some authors [30,31] proposed histogram-based tone-mapping curves, the slopes of which are dependent on the measured probability from the distribution of luminances.

The performance of local tone-mapping methods also depends on the local structure of the image content. Detail preservation is performed by using edge-preserving filters or multi-resolution analysis. In earlier works on tone-mapping [24], Gaussian filtering was used for extracting the local luminance structures. However, these approaches resulted in halos for large gradient edges. Other approaches make use of pyramid filtering structures [13,21,25]. Still other approaches [20] use edge preserving filters (e.g., bilateral filter) to avoid the halo artifacts typically seen with Gaussian filters. However, with these approaches, problems were encountered at steep and smooth edges, where banding artifacts were introduced. There are also approaches [28] that employ the concept of high-quality edge-preserving filtering on the already established tone-mapping [13].

In the evaluations performed in [3,9], the global tone-mapping algorithm of Reinhard et al. [13] consistently ranks high, preserving contrast well and producing perceptually pleasing images. Farbman et al. [28] have proposed an extension of the Reinhard algorithm with local tone-mapping concepts. Therefore, in this paper, we consider the methods of Reinhard et al. [13] and Farbman et al. [28], which are representative of the classical SOTA.

Most recently, deep-learning-based algorithms [32,33,34,35,36,37] for tone-mapping, as well as algorithms that jointly perform tone-mapping with denoising [38], have been proposed.

### 2.2. HDR Imaging and Object Detection

Some works specifically focus on HDR for the purpose of object detection. In [39], the authors perform a study in which they evaluate classical methods for tone-mapping, with respect to object detection accuracy on the tone-mapped 8-bit content. Their evaluation is performed on an available limited HDR–SDR data set, that is not specifically traffic-related and, therefore, does not specifically cover cases that are challenging for ADS. In the study cited above, the authors are investigating the usefulness of HDR data over SDR data in improving object detection in general. Their finding is that, in very challenging lighting conditions, using HDR content over SDR content helps in improving object detection accuracy. In [40], the authors propose a methodology for obtaining a pseudo HDR data set that can be used for training and re-training object detectors on HDR data of high bit-depth. They also investigate the performance of several object detectors when they are trained on HDR data. Their results show that the object detectors have similar performance regardless of whether they are trained on SDR or HDR data when the source is HDR content and the main difference arises from whether the content is non-linearly tone-mapped to 8 bits or used directly. In [41], the authors propose a deep learning model that selects the best exposure and is trained with an object detector in an end-to-end training. An end-to-end training, where both object detection and tone-mapping are optimized, is also proposed in [42].

Although there is at least one study that performs object detection on HDR content, the main SOTA object detection algorithms on which improvements are constantly being made are trained on 8-bit content. A training approach that uses a lightweight CNN architecture for tone-mapping with the intention, for the first time, of increasing the detectability of VRUs in reconstructed 8-bit content, is proposed in [2]. The results of a comparison with one of the classical state-of-the-art algorithms for tone-mapping show that this approach, by targeting the important aspects of tone-mapping in various challenging conditions and by performing a region-of-interest (ROI) selective training, succeeds in increasing the detectability of the VRUs in both challenging and non-challenging cases.

### 2.3. Demosaicing

Demosaicing is defined as the reconstruction of a full-color (or multi-dimensional) image from a two-dimensional array of sensors overlaid with a color filter array (CFA). The most exploited CFA in camera imaging sensors is the Bayer CFA [43]. In the last few decades, a tremendously large amount of research has been performed on CFA demosaicing design and co-design [44,45,46,47,48], and the topic of demosaicing itself (survey studies [49,50,51,52,53]) ranges from state-of-the-art classical methods [54,55,56,57,58,59] to a wide palette of deep-learning-based methods [60,61,62,63,64,65].

There are studies [51,61] that show that, among the classical methods, the algorithms that perform directional interpolation, such as [56,57,59], achieve high-quality performance and are considered to belong among the state-of-the-art classical demosaicing methods. Due to the enormous amount of research in this field, achieving high-quality demosaicing is no longer a problem and the obtained quality between the recently developed methods is similar. Consequently, most recent deep-learning-based demosaicing methods approach the problem of demosaicing in a joint manner with denoising [62,64,66,67].

It is well known in the scientific community that building a joint model to solve two problems that co-exist and are co-dependent is the optimal way to approach the two problems simultaneously.

### 2.4. Novelty of the Performed Research

SOTA algorithms for tone-mapping, object detection and demosaicing mainly exist and have progressed independently from each other. With the performed research, which builds upon the research presented in [2], we address the common ADS problem they are trying to solve, which is improving object detection in challenging traffic scenes. With the proposed research, we attempt to increase the detectability of the traffic-related objects in traffic scenes with varying degrees of illumination, contrast, weather and daylight ambient conditions by performing optimal tone-mapping and demosaicing on HDR content.

We start by defining two research hypotheses.

Our first research hypothesis is that by performing optimal tone-mapping with noise suppression while focusing on ADS object detection, we will improve the detectability of traffic-related objects and pedestrians compared to two fundamental cases: when SDR content is used and when state-of-the-art demosaicing and tone-mapping (TM) algorithms are applied in a sequential pipeline.

The second research hypothesis is that by using the raw (mosaiced with Bayer CFA) HDR image as input instead of the full-color, full-dynamic-range (e.g., 12 bits/16 bits per color channel) representation of it, the joint demosaicing and tone-mapping with noise suppression can be performed in an optimal manner and we can achieve the same or similar performance and at the same computational cost as with the sequential pipelines from the first hypothesis.

For this purpose, we model two neural networks (CNNs) with similar architectures, complexity and training. The first network, “CNN for TM”, is devised solely for tone-mapping where a full-color, full-range HDR input is used. This network builds upon the architecture and the methodology presented in [2]. This network is used in the sequential demosaicing and tone-mapping pipeline. The second neural network, “CNN for joint DM and TM”, is devised to jointly perform both demosaicing and tone-mapping.

With the two proposed CNN architectures, we propose tone-mapping optimized to increase the detectability of objects in traffic-related scenes with challenging conditions. The first novelty of the performed research is that for the first time, to the best of our knowledge, a CNN-based algorithm for joint demosaicing and tone-mapping has been proposed. Both CNN architectures are additionally trained to suppress noise and reconstruction artifacts. To test the hypotheses, we introduce a second novelty, which is an extensive evaluation study performed on real traffic-related content. The evaluation is performed with respect to ADS object detection accuracy and reconstruction (tone-mapping and demosaicing) quality. With this study, we evaluate the impact of the proposed approach on the detection of vulnerable road users (VRU) and traffic-related objects (such as traffic lights and traffic signs) important for road safety. Object detection performance is evaluated by using well-known object detectors, applied on the reconstructed 8-bit content, covering a large number of scenes with challenging (i.e., boundary) cases. Additionally, as part of the evaluation study, we perform a qualitative analysis and comparison of the joint processing pipeline with the sequential processing pipelines. The evaluation experiments compare the CNN for joint DM and TM, the sequential pipeline of state-of-the-art demosaicing and the CNN for TM and the sequential pipelines of competing state-of-the-art demosaicing and tone-mapping algorithms from the literature. The evaluation is performed on a novel SDR/HDR test data set constructed for this purpose.

### 2.5. ExpandNet

**Figure 2:**
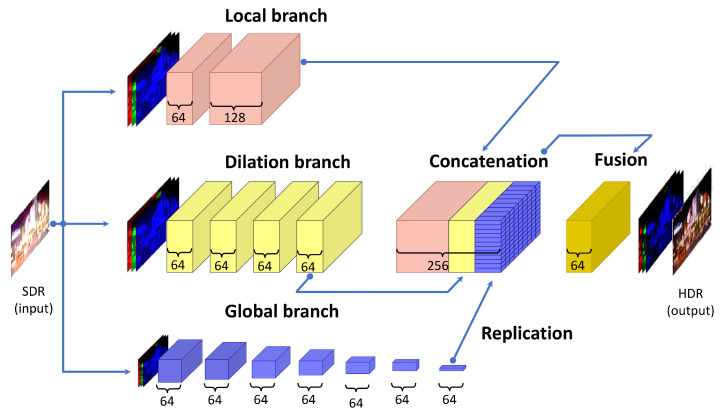
ExpandNet [68] architecture. The presented architecture is used as the starting point for the approach presented in [2], on which we build the proposed CNN for TM and CNN for joint DM and TM. The image is recreated and uses a similar graphic design as the original image source presented in [68].

Here, we briefly describe ExpandNet [68] (implementation: [69]), a deep learning algorithm for inverse tone-mapping. Its original architecture was used as the starting point for the training approach presented in [2], on which we build the proposed CNN for TM and CNN for joint DM and TM. The end-to-end architecture design of ExpandNet [68] is presented in Figure 2. The ExpandNet algorithm converts the input SDR image into content of higher dynamic range. The network architecture consists of three branches (each a CNN): global, local and dilation.

The global branch accounts for the higher-level image-wide global features and the overall appearance of the output. With its large receptive field, it covers an input image of 256×256×3 pixels downsampled to 1×1×3 through seven layers. For each layer, there are 64 features and the downsampling is performed with a factor of 2. The size of the convolutional kernel for all layers except for the last one is 3×3×64. For the last layer, the convolutional kernel is of size 4×4×64. If the input image is larger than 256×256, it is downsampled to 256×256 prior to feeding the global branch of the network with the image data.

The local branch accounts for preserving the local structure, the high spatial frequencies and the neighboring features. Its receptive field is 5×5 pixels and it consists of two layers. The convolutional kernel is 3×3. The first layer has 64 feature maps and the second layer has 128 feature maps.

The dilation branch accounts for preserving the medium range frequencies. It has a receptive field of 17×17 pixels, uses dilated convolutions (for increasing the receptive field) of size 2 and consists of 4 layers, where each layer has 64 features.

The final layers of the three branches are first concatenated and then fused into one additional layer of 64 feature maps. The output of this final convolutional layer and, therefore, the ExpandNet algorithm, here referred to as the “HDR image”, is a converted image of a higher dynamic range than the input SDR image.

The loss function is a combination of L1 distance and a cosine similarity measure between the predicted image and the ground-truth image. The cosine similarity term is used to ensure fidelity of the colors in every pixel. As the authors of [68] explain, the cosine similarity term measures how close two vectors are by comparing the angle (color hue difference) between them regardless of the magnitude (brightness). To obtain color fidelity during training, the difference in the vector directions in the 3D RGB space of the corresponding two pixel vectors is quantified with the cosine similarity metric and added to the loss function. The loss function is shown in Equation (1). We use the same annotation as in the original ExpandNet paper [68].

(1) $$l_{i}={∥{\tilde{I}}_{i}-I_{i}∥}_{1}+\lambda \left(1-\frac{1}{k}\underset{j=1}{\sum^{k}}\frac{{\tilde{I}}_{i}^{j}\cdot I_{i}^{j}}{{∥{\tilde{I}}_{i}^{j}∥}_{2}{∥I_{i}^{j}∥}_{2}}\right)$$

In Equation (1), $l_{i}$ represents the loss contribution of the *i*th image from the training data set, with which, for one epoch, the overall loss over the complete training data set is being updated. The predicted image is represented by ${\tilde{I}}_{i}$ and the ground-truth image is represented by $I_{i}$. The two images are compared for every RGB pixel, the indices for which in Equation (1) are represented by j. There are k pixels in every image of the data set. The λ parameter controls the influence of the cosine similarity term on $l_{i}$.

Since tone-mapping is conceptually the same as inverse tone-mapping, the same basic network architecture with swapping the places of the input and the output data and appropriate retraining, can be used to deal with both tone-mapping and inverse tone-mapping problems. Starting from this idea, like the approach presented in [2], for the proposed CNNs, we are using modified versions of this architecture and a similar training concept. The loss and the activation functions, the convolutional kernels and the background CNN theory used in the proposed CNNs are the same as for the ExpandNet algorithm and are described in detail in the complete architecture design description given in [68].

## 3. Proposed Algorithms

In this section, we first give a short introduction to the processes specific for image acquisition. This is in order to perform training augmentation on the training data used for the proposed CNNs. In addition to accounting for diverse illumination and contrast conditions of the scene, we also account for the physical processes during image acquisition and the internal camera processing. We then proceed with the details of the proposed CNNs in the following order:

- The devised architectures of the proposed CNNs, based on the architecture of the ExpandNet [68] network;
- The concepts of the training and inference for the proposed CNNs;
- The details of the training and the validation data sets;
- The details of the augmentation procedures as part of the training methodology for the proposed CNNs.

### 3.1. On Simulating the Effects from the HDR Image Acquisition Processes

The acquisition of HDR images with a single-shot HDR camera follows the same principle as every standard image acquisition process.

In a standard image acquisition process, the light from the scene passes through the camera lenses, after which it is focused and projected on the image sensor. The image sensor consists of an array of sensing nodes, the number of which defines the resolution of the output image. The sensing node converts the incoming photons into electrical signals. Each sensing element is overlaid with a color filter. The color filters are arranged into a planar mosaic structure called a color filter array (CFA). The CFA adds wavelength specificity to the image sensor, which without the CFA will detect light in an achromatic manner. The conversion of light depends on the structure, the sensitivity of the sensor material and the wavelength of the incoming light. During conversion, there is photon noise, which interferes with the useful signal. The electrical signal obtained from each sensing node is passed through read-out circuits and analog-to-digital (AD) conversion modules, which are attached to every sensing node of the sensor. The AD converters discretize the analog signal depending on their resolution and convert it into a digital form. The process of conversion from analog to digital form is known as quantization. In order to account for the effects from the image acquisition process on the image data, it is necessary to simulate this process during training of the proposed CNNs. In addition to performing data augmentation (where we account for diverse illumination and contrast conditions in the scenes) during training, we additionally simulate:

- The acquired HDR training data (by applying the ExpandNet algorithm for inverse tone-mapping on an SDR data set consisting of traffic scenes annotated for traffic-related objects);
- The existence of the CFA (by creating mosaics, i.e., Bayer CFA, of the input training images to the CNNs);
- The presence of photon noise (by the application of noise with a Poisson distribution on the simulated mosaiced data);
- The AD conversion process (by applying quantization with a predefined bit-depth parameter higher than 8 bits per channel);
- The internal camera processing (by applying demosaicing with the use of a classical state-of-the-art algorithm [56]).

### 3.2. Proposed CNN for TM

Unlike ExpandNet [68], the proposed CNN for TM accepts full RGB HDR images as input and performs conversion into the low dynamic range. It produces a tone-mapped-HDR-image (8 bits per channel) of the same resolution as the input. In what follows, we present the architecture of the proposed network and the workflow for training and inference.

#### 3.2.1. Architecture

Our modification of the Expandnet [68] consists of just two branches, the local and the global.

**Figure 3:**
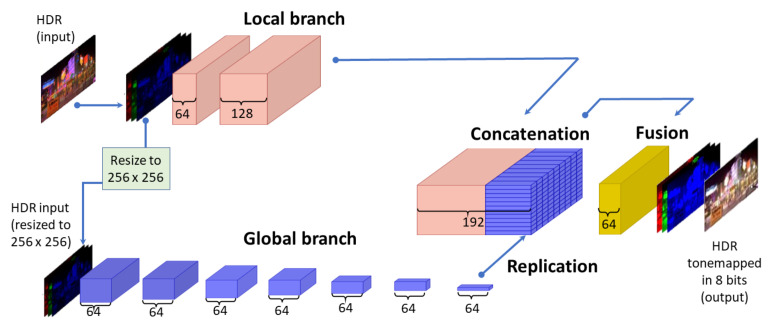
Architecture for the proposed CNN for TM. The presented architecture is modified from the approach presented in [2], which uses the original ExpandNet architecture [68] as its basis. The graphic design is inspired by the original image source presented in [68].

Thus, compared to the original ExpandNet [68], as it can be seen in Figure 3, the dilation branch is removed. In the case of ExpandNet [68], the results from the ablation study, where each of the branches is separately removed and then the performance is analyzed, show that the low and high spatial frequencies, as well as the overall image appearance, are largely preserved with the use of just the global and local branches.

In the proposed CNN for TM, by not using the dilation branch from the ExpandNet algorithm, we aim to decrease the computational cost, while at the same time, preserve the overall appearance of the reconstructed content. In this manner, we make a small compromise on the image sharpness in order to obtain lower computational cost.

The input to the local branch is the full RGB HDR image, while the input to the global branch is a resized version (256×256×3) of the full RGB HDR input image. The structure of the local and the global branches, the activation and the loss functions and the convolutional kernels are same as for ExpandNet. After concatenating the final layers of the separate branches, 192 feature maps are fused into one additional layer consisting of 64 feature maps, out of which the tone-mapped-HDR-image is produced.

**Figure 4:**
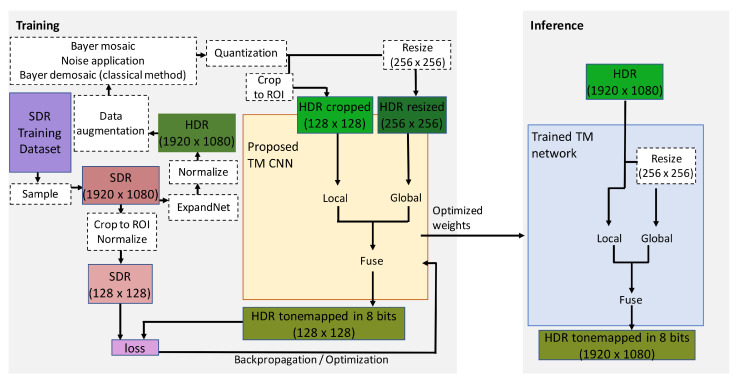
Workflow for the proposed tone-mapping network: training and inference. The graphic design for the illustration was inspired by [68].

The workflow for the proposed CNN for TM is presented in Figure 4.

#### 3.2.2. Training and Inference

During training, an SDR ground-truth image sample is selected from the training data set. The SDR ground-truth image is converted to a high dynamic range with ExpandNet [68] and an HDR representative is obtained. The HDR representative is processed through the data augmentation procedures and the procedures for simulating the HDR acquisition process. Part of the HDR representative is selectively cropped (128×128×3) in the ROI of the traffic-related objects and is propagated through the local branch. The complete HDR representative is first downsampled to 256×256×3 and then it is propagated through the global branch. The output from the fusion branch is compared to the corresponding ROI cropped part from the SDR image sample, and the loss is calculated. Then, the weights and the overall loss are updated. The proposed CNN model for TM uses the same architecture as the approach presented in [2], differing only in the image size of the input data on which it is being trained. The image samples also belong to the same training data set. The only difference is that for the training of the new model, the image samples have been resized to 1920×1080×3, whereas in the approach of [2], the training image samples are of size 1280×720×3. By doing this, we intend to match the resolution and the actual size of the objects in the image content from the test data set. The model of the proposed CNN for TM was trained for 3000 epochs.

During inference, the network accepts full RGB HDR image data of resolution 1920×1080×3 normalized in the range of [0, 1]. For the global branch, this image is resized to 256×256×3 pixels. The tone-mapped-HDR-image is of same resolution as the input full-color, full-range, HDR image.

### 3.3. Proposed CNN for Joint DM and TM

The proposed CNN for joint DM and TM accepts raw HDR (CFA mosaiced with the Bayer pattern) image as input and produces a full-color, 8-bit, tone-mapped-HDR-image, as output. In what follows we present the architecture of the proposed CNN for joint DM and TM, and the workflow for training and inference.

#### 3.3.1. Architecture

**Figure 5:**
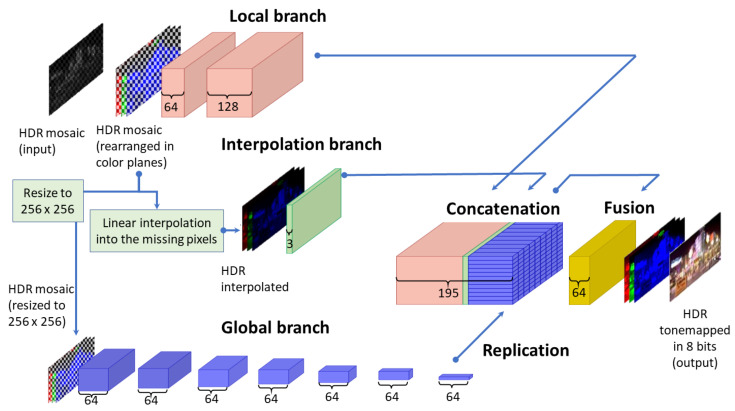
Architecture of the proposed CNN for joint DM and TM. The presented architecture is modified from the approach presented in [2], which is based on the ExpandNet architecture [68]. The graphic design is inspired by the original image source presented in [68].

The architecture, as can be seen from Figure 5, is an extended version of the architecture presented in Figure 3. It consists of three branches: the local branch, the global branch and the interpolation branch. In the proposed CNN for DM and TM, we use the local and global branches in the same manner and for the same reasons as in the proposed CNN for TM. The input of the local branch is the mosaiced image rearranged in three color channels (depending on the version of the Bayer pattern), while the input to the global branch is a resized version (256×256×3) of the input image to the local branch. The interpolation branch consists of one layer for the three color channels of the linearly interpolated mosaiced image, which is then propagated to the further layers of the complete neural network. We choose linear interpolation due to its simplicity and under the assumption that the neural network, with the other two branches, is capable of learning how to perform more complex high-quality interpolation. Our aim is to prove the research hypotheses with simple solutions rather than applying sophisticated and more complex interpolation methods. The linearly interpolated image is a first, simple estimate of the HDR demosaiced image. It serves as a prior image estimate for the further processing and weights adjustment. Normally, the linear interpolation of a CFA mosaiced image produces output with color aliasing artifacts around the edges. This is a disadvantage of the interpolated image to be used as a first estimate of the full RGB HDR input. However, it is expected that the network during training, by minimizing its loss function, would be capable of learning to deal with the problem of artifact introduction due to incorrect interpolation in the initial estimate. Moreover, it is expected that when an interpolated image is available, the network will efficiently learn to make a distinction between zero values due to missing pixels in the rearranged three-channel mosaiced image and the zero values because of absence of light in the pixel of interest. It is also expected that in case of demosaicing artifacts in the interpolated HDR image, they will occur in the output tone-mapped image as well, which through comparison with the ground-truth artifact-free SDR image, will increase the loss measure in its two terms. In that way, the existence of the negative feedback loop during training for the introduced artifacts and errors is ensured.

**Figure 6:**
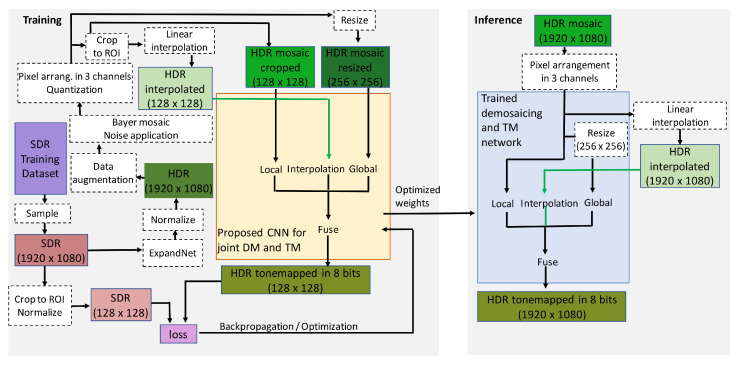
Workflow for the proposed CNN for joint DM and TM: training and inference. The graphic design for the illustration was inspired by [68].

The used activation and loss functions and the structure of the local and the global branches are the same as for ExpandNet. After concatenating the final layers of the three separate branches, 195 feature maps are fused into one additional layer consisted of 64 feature maps, out of which a demosaiced, full-color, tone-mapped HDR image output is produced. The workflow for the proposed CNN for joint DM and TM is presented on Figure 6.

#### 3.3.2. Training and Inference

During training, the SDR ground-truth image sample selected from the training data set is first converted to high dynamic range with ExpandNet [68] and an HDR representative is obtained. The HDR representative is processed through the data augmentation procedures and the procedures for mosaicing, noise application and quantization. For feeding the interpolation branch, the mosaiced version of the HDR representative is interpolated in the missing pixels with linear interpolation. Part (128×128×3) of the mosaiced (after rearrangement into three channels) HDR representative is selectively cropped and propagated through the local branch. The corresponding part (128×128×3) is selectively cropped from the linearly interpolated HDR image and is sent to the interpolation branch. Through the global branch, a down-scaled version (256×256×3) of the mosaiced (rearranged into three color channels) HDR representative is propagated. The image output of the fusion network branch is compared to the corresponding ROI cropped part of the SDR ground-truth image sample, and the loss is calculated. The overall loss and the trainable weights are accordingly updated. The model of the proposed CNN for joint DM and TM was trained for 1900 epochs.

During inference, the network accepts a mosaiced (Bayer CFA) HDR image of size 1920×1080×1 normalized in the range of [0, 1]. The mosaiced image is rearranged into three channels. For the global branch, this image is resized to 256×256×3. The full-color tone-mapped HDR image output is of resolution 1920×1080×3.

### 3.4. Training and Validation Data Sets

In absence of available SDR–HDR data that can be used in research topics related to ADS, for the purpose of training the proposed networks, we use an SDR data set consisting of large amount of diverse traffic/road scenes annotated for traffic-related objects. From the images of this data set, with the use of ExpandNet [68], we simulate the corresponding HDR equivalents. The data set was formally released in 2021 and is known as “Berkeley Driving Dataset 100K” [70,71]. Its implementation is available at [72]. For the purposes of this research, we use the image subset for object detection, which consists of roughly 4300 images. This subset of the complete data set

- Is annotated for 10 object categories: car, traffic sign, traffic light, person, truck, bus, bike, rider, motor and train;
- Consist of scenes with diverse weather conditions: rain, snow, sunlight, cloudy weather;
- Consists of scenes captured at different times of day: nighttime, daytime, dawn/dusk;
- Consists of scenes in various locales: city streets, residential areas, highways.

Despite the fact that this dataset abounds with the content of interest, similar to the approach presented in [2], we use the attached annotations for automatic selective cropping during the performed training, i.e., to crop the part of the image where most of the objects of interest are located. For training, we use 3657 image and for validation 550 images. This image subset was the only one available from the whole “Berkeley Driving Dataset 100K” that is completely annotated for 10 object categories. Normally, a subset of more than 4300 image samples will contribute to better generalization in the training process. However, the content abounds with cases from diverse traffic situations, both very challenging and easy for the ADS object detection. Moreover, we apply augmentation procedures to additionally account for different times of the day, difficult illumination and contrast conditions, as well as the presence of noise. By doing this, we largely increase the diversity of the data set used for training, and we consider that roughly 4000 image samples in different representations over more than 1500 epochs for the both CNNs is sufficient to obtain reconstructed content of satisfying quality.

Unlike the approach presented in [2], we do not use the image samples from this data set in their original resolution. In order to match the resolution of the real HDR test data set (presented in Section 4.1), we resize the images from the training SDR data set (the original resolution is 1280×720) to 1920×1080 and also update the annotation boxes with the corresponding resize factor.

### 3.5. Training Methodology

Here, we briefly describe the training procedures (which also serve as augmentation procedures) that we apply to simulate diverse conditions of the HDR image acquisition processes. Furthermore, we describe the procedures for data augmentation in order to account for diverse contrast and lighting conditions. For more details about the procedures, we direct the reader to [2]. The input HDR representatives, on which the training procedures are applied, are obtained by applying the ExpandNet algorithm [68] for inverse tone-mapping on the SDR image samples from the training data set. Unlike the approach presented in [2] and in order to be consistent in simulating the camera acquisition process, for each simulated HDR input sample into the training, we perform the procedures in the following order:

- Apply data augmentation (contrast and color augmentation) to account for different scenes;
- Apply Bayer CFA mosaicing;
- Simulate photon noise;
- Simulate quantization;
- For the proposed CNN for TM, perform demosaicing with a classical SOTA algorithm [56] to simulate the internal camera processing. This procedure is not applied during training of the proposed CNN for joint DM and TM.

#### 3.5.1. Pre-Processing

Due to the low brightness of the training data set nighttime scenes, in order to train the proposed CNNs to improve the objects visibility in such scenes, we perform pre-processing on the contrast of the dark ground-truth images. We do that by increasing the exposure of the nighttime SDR samples from the training data set. This is done with the following equation:

(2) $$I_{\mathrm{enh}.\mathrm{night},\;i}={\left(I_{\mathrm{orig}.\mathrm{night},i}^{\delta }\cdot 2^{s}\right)}^{\frac{1}{\delta }}$$

In Equation (2), $I_{\mathrm{orig}.\mathrm{night},i}$ refers to the original nighttime SDR image sample, where i is the number of the image sample from the training data set, and $I_{\mathrm{enh}.\mathrm{night},\;i}$ refers to the obtained enhanced SDR version of the nighttime sample. The exponential factor δ=2.2 is used to account for the camera response function, while the exponential factor s∈[0.5,1.5] is used to simulate the exposure increase in stops.

#### 3.5.2. Data Augmentation

As part of the data augmentation, we apply contrast augmentation and color temperature augmentation techniques in sequence.

##### Contrast Augmentation Procedures

With the contrast augmentation techniques, by creating realistic scenes from the training data set, we aim to train the proposed CNNs to become robust on diverse contrast conditions, spanning from very challenging (e.g., entering a tunnel, or the occurrence of very bright parts against very dark shadows in the same scene) to less challenging and normal conditions (where the objects have good visibility). We perform this by changing the contrast in range of five orders of the magnitude between the brightest and the darkest parts of the scene.

In a same approach as that presented in [2], we apply three different techniques for the contrast augmentation. These are:

- Gamma expansion (applied either on the luminance channel or on every color channel);
- Sigmoidal contrast stretching [73] (applied either on the luminance channel or on every color channel);
- Selective contrast degradation [2] (to simulate very difficult conditions) applied on the luminance channel.

##### Color Temperature Augmentation Procedures

With the color temperature augmentation, as it is the case in the approach presented in [2], we ensure the existence of diverse realistic daytime ambient light conditions and use this to train the proposed CNNs to obtain color-balanced output, especially in challenging (difficult) conditions. We apply the color temperature changes as described in [74], in the range from 2000 K to 10,000 K.

#### 3.5.3. CFA Mosaicing

To simulate the CFA with which the camera sensor is overlaid, we perform RGGB Bayer mosaicing with three orthogonal subsampling functions $m_{i}\left(x\right)$ taking values 1 or 0, if a color is supposed to be present at the pixel location or not, as shown in Equation (3).

(3) $$H_{\mathrm{CFA}}\left(x\right)=\sum_{j}H_{j}\left(x\right)m_{j}\left(x\right)$$

In Equation (3), $H_{j}\left(x\right)$ is the intensity from the j-th color component of the simulated HDR image H at the pixel location given with coordinates x, while $H_{\mathrm{CFA}}$ is the obtained CFA mosaiced HDR image and $H_{\mathrm{CFA}}\left(x\right)$ is the corresponding intensity at the pixel location given with x.

#### 3.5.4. Noise Application

The photon (shot) noise that interferes with the useful signal, arises in the camera acquisition process from the uncertainty of the quantity of photons, impacting the sensing node in a short time interval, under unchanged and constant illumination. This process is coupled with uncertainty of the quantity of electrons excited within the sensor semiconductor for the same time interval. This is a process that is signal-dependent and it follows a Poisson distribution. We simulate a CFA mosaiced noisy HDR image $H_{\mathrm{CFA},\mathrm{noise}}$ by applying the Poisson distribution on the pixel intensities of the obtained CFA mosaiced HDR image in the following manner:

(4) $$H_{\mathrm{CFA},\mathrm{noise}}\left(x\right)∼\mathrm{Pr}\left(H_{\mathrm{CFA}}\left(x\right)\right)$$

(5) $$\mathrm{Pr}\left(k\right)=\frac{e^{-\lambda }{\left(\lambda \right)}^{k}}{k!}$$

where ∼ means “is distributed as”, k is the number of occurrences (i.e., number of photons measured with the sensor element), which in our simulated case corresponds to the pixel intensity of $H_{\mathrm{CFA}}$, at the pixel with coordinates x, *e* is the Euler’s number and λ is the expected rate of occurrences. For larger values of λ, the Poisson distribution approaches a Gaussian distribution. Therefore, the noise in general is commonly modeled with a Gaussian distribution, too.

In our realistic noise simulation, the λ parameter changes from 0.2% (simulating low-level noise) to 20% (simulating high-level noise) of the maximum pixel intensity.

#### 3.5.5. Quantization

The process of mapping continuous values (an infinite set of values) of a signal to a finite (countable) set of discrete values is called quantization. The process of quantization happens immediately after the analog-to-digital (AD) conversion. In our simulation, the quantization is applied immediately after the application of noise to the mosaiced HDR image. For example, if the AD converter is of 24 bits, this means that there will be $2^{24}$ = 16,777,216 levels, with 0 representing the darkest level and 16,777,215 being the brightest level. The quantization process introduces a quantization error, which happens due to rounding and truncation. The quantization error is equal to the difference between the actual input value and its quantized value. The presence of quantization errors is modeled with quantization noise, which is of much lower intensity and influence on the captured data compared to the photon noise. To simulate high bit-depth HDR data, quantized to 24 bits, we perform the quantization in the following manner:

(6) $$H_{\mathrm{CFA},\mathrm{noise},q}\left(x\right)=\min\left(\max\left(\frac{\left\lfloor \left(2^{q}-1\right)\cdot H_{\mathrm{CFA},\mathrm{noise}}\left(x\right)\right\rfloor }{\left(2^{q}-1\right)},0\right),1\right)$$

where $H_{\mathrm{CFA},\mathrm{noise}}\left(x\right)$ is the pixel intensity of the CFA mosaiced and noisy HDR image after being normalized in the range [0, 1], q=24 is the bit-depth to which we aim to quantize the image, $\left\lfloor *\right\rfloor$ is the rounding operator, $\min\left(*,1\right)$ is an operator that takes the minimum of two numbers and clips the signal to the maximal value (1 in the case of a normalized signal) and $\max\left(*,0\right)$ is an operator that takes the maximum of two numbers and ensures that there is no negative value in the signal.

#### 3.5.6. Demosaicing

In order to simulate the demosaicing from the internal signal processing pipeline of the camera on the augmented, mosaiced and noisy HDR images simulated during training, we apply the classical SOTA algorithm of Menon et al. [56]. The demosaicing approach presented in [56] performs an effective directional interpolation, where the most suitable interpolation direction for the blue and the red color components is made with an a posteriori decision based on the reconstructed green component. When such a decision is made and the color components are initially reconstructed, they are further refined based on the local variation in the color differences along the horizontal and the vertical directions.

#### 3.5.7. Importance of the Applied Pipeline of Sequential Procedures Mosaicing-Noise Application–Quantization-Demosaicing

In simulating the image acquisition process, it is important to apply the described procedures in the presented order:

- Mosaicing;
- Application of noise with Poisson distribution;
- Quantization;
- Demosaicing.

**Figure 7:**
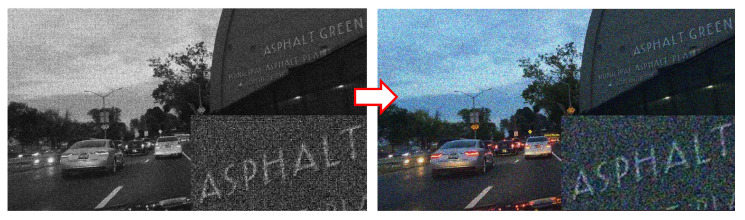
The appearance of noise with demosaicing and noise artifacts when the noise is applied before demosaicing, as happens in reality. Left: Bayer CFA mosaiced image with noise with Poisson distribution. Right: the image on the left demosaiced with the algorithm presented in [56]. Note the complex structure and the severity of the noise artifacts in the demosaiced image. After demosaicing, the noise appears with a more complex structure than the initial Poisson distribution in the mosaiced image. It is color correlated and it is dependent on the local spatial activity of the image content.

When the noise is applied on the CFA mosaiced image and then the image is demosaiced, the noise has a granular structure, is color correlated and is dependent on the local spatial activity, thus creating noise artifacts (see Figure 7). Therefore, it is hard to state that at this point, this complex noise structure follows the Poisson distribution.

For this reason, the reconstruction algorithms (among which we consider the tone-mapping algorithms, too) typically handle the problem of noise and demosaicing artifacts by approaching it in a joint manner [66,75,76]. Following the same practice:

- For the proposed CNN for TM, we approach the problem of tone-mapping and noise and artifact suppression in a joint optimal way;
- For the proposed CNN for joint DM and TM, we approach the problem of demosaicing, tone-mapping and noise and artifact suppression in a joint optimal way.

## 4. Evaluation

In this Section, we describe the test data set, select the SOTA algorithms for comparison and define the metrics and the methodology for the performed quantitative and qualitative analyses.

### 4.1. Test Data Set

The real test data set, which is the same test data set as that used in [2], consists of 371 video frames extracted from synchronized HDR–SDR video sequences. The HDR data consists of raw (Bayer CFA mosaiced) frames and is acquired with a real HDR camera (using the Sony IMX490 sensor) with a dynamic range of 120 dB. The resolution of the frames is 1920×1080. The extracted frames (images) consider scenes:

- From different times of day: nighttime, dawn/dusk, daytime;
- With different lighting conditions: very dark scenes, very bright scenes and scenes with strong lights in the background;
- From traffic jams and from lower frequency traffic;
- Abundant with VRUs and objects crucial for road safety.

The extracted images are annotated for the object classes: “person”, “colored traffic light” and “traffic sign” (danger, mandatory, prohibitory and stop sign). Annotated in the extracted images, there are:

- 1477 objects belonging to the class “person”;
- 998 objects belonging to the class “traffic sign”;
- 357 objects belonging to the class “colored traffic light”.

Additionally, information about the scene was added in the annotation files, addressing the following aspects:

- The time of day;
- Whether there is sun glare or strong lights present in the scene;
- Whether the captured scene is too bright, too dark, or the lighting conditions are normal;
- Whether or not there is occlusion of the objects or the VRUs of interest;
- Whether or not there is motion blur (caused by a fast change in the motion of the platform on which the cameras are positioned or because the VRUs are moving too fast) in the content visible to the annotator;

We use this information in order to split the data set into difficult/challenging scene cases and easy/non-challenging scene cases. The criteria considered to classify a scene as difficult/challenging are:

- Existence of strong lights or sun glare in the scene;
- It is a night scene;
- Most of the relevant objects are occluded and hence hard to detect;
- The scene is either too dark or too bright;
- There is motion blur.

We use the created data set to test the proposed CNNs in comparison to the SOTA algorithms and the SDR content with the metrics and the methodology explained in Section 4.3.

### 4.2. Methods for Comparison

Since the main scopes of this research are tone-mapping and demosaicing approaches, we evaluate the proposed CNNs against sequential pipelines of state-of-the-art demosaicing and tone-mapping algorithms.

As representative of the state-of-the-art demosaicing methods and due to its ease of use, we select the algorithm of Menon et al. [56] (here referred to as “Menon et al.”).

Based on previously performed analyses [3,9,38], we select the algorithm of Reinhard et al. [13] (here referred to as “Reinhard et al.”) as representative of the state-of-the-art classical global tone-mapping approaches and the algorithm of Farbman et al. [28] (referred to as “Farbman et al.”) as representative of the state-of-the-art classical local tone-mapping approaches. Since the deep learning tone-mapping algorithms are in an emerging phase and not all of them have publicly available implementations, we select the algorithm of Vinker et al. [34] (referred to as “DL HDR TM”, which stands for deep learning HDR tone-mapping) as representative of the deep-learning class of algorithms for tone-mapping. Prior to applying the TM algorithms, Reinhard et al., Farbman et al., the DL HDR TM and the proposed CNN for TM, we apply demosaicing of the real HDR data with the algorithm of Menon et al. [56]. For the proposed CNN for joint DM and TM, we do not perform prior demosaicing and feed the CNN with the raw HDR data, normalized in the range [0–1].

The tone-mapping settings for the classical methods were optimized to produce the best results (in terms of PSNR and SSIM) on the training data set. We use the same optimized parameters for the real test data set. There are no tone-mapping parameters to be adjusted for the algorithm of Vinker et al. [34], and the neural network (publicly available at [77]) is applied as it was trained by the authors.

### 4.3. Metrics and Methodology for Evaluation

For the evaluation study, we perform quantitative and qualitative analyses. For the quantitative analysis, we perform object detection evaluation over the real HDR–SDR images from the test data set, on the content obtained with the proposed CNNs and the SOTA methods from the literature, as well as for the SDR content. For the qualitative analysis, we first perform visual comparison on the object detection results and then we continue with qualitative analysis on several aspects of the quality of the tone-mapping and the demosaicing performance.

#### 4.3.1. Quantitative Analysis

∗ The object detection evaluation is performed with using the following object detectors:
  - Yolo v3 [1] for the classes “person”, “stop sign” and “traffic light” (only detection on colored traffic lights is evaluated, as being the most relevant in tone-mapping and for road safety).
  - Yolo v2 version, trained for three subcategories of the class “traffic sign” (“danger”, “mandatory”, “prohibitory”), with an analysis presented in [78] and implementation publicly available at [79].
∗ The object detection performance is analyzed for each of the three classes: “person” (P), “colored traffic light” (TL) and “traffic sign” (TS). For the class “traffic sign” (TS), we consider the three subcategories of traffic signs in combination with the “stop sign”.
∗ From the available metrics for object detection, we use:
  - F${}_{2}$ score: because it is a metric that combines precision and recall, while it penalizes the “missed detections” (False Negatives, i.e., FN) more than the false positives (FP) and unlike the symmetric F${}_{1}$ score, it gives more weight to the recall than to the precision;
  - True Positive Rate (TPR or recall), as a metric for correct detections;
  - False Negative Rate (FNR), as a metric for missed detections;
  - False Positives Per Image (FPPI): a metric that is calculated as the average number of false positives (FP) over all images in the test data set.
  The F${}_{\beta }$ score, where $\beta \in \left\{0.5,1,2\right\}$, is shown in the following equations. In general, the F${}_{\beta }$ score, as a function of the number of True Positives (TP), number of False Positives (FP) and number of False Negatives (FN), is given by:
  (7) $$F_{\beta }=\frac{\left(1+\beta^{2}\right)\cdot \mathrm{TP}}{\left(1+\beta^{2}\right)\cdot \mathrm{TP}+\beta^{2}\cdot \mathrm{FN}+\mathrm{FP}}.$$
  When β=2, F${}_{\beta }$ becomes F${}_{2}$ score:
  (8) $$F_{2}=\frac{5\cdot \mathrm{TP}}{5\cdot \mathrm{TP}+4\cdot \mathrm{FN}+\mathrm{FP}}.$$
  As a function of precision and recall, the F${}_{\beta }$ score in general can be calculated in the following manner:
  (9) $$F_{\beta }=\frac{\left(1+\beta^{2}\right)\cdot \mathrm{precision}\cdot \mathrm{recall}}{\beta^{2}\cdot \mathrm{precision}+\mathrm{recall}}.$$
∗ The object detection evaluation is performed in two ways:
  - By changing the detection thresholds in the range [0–1] of the yolo algorithms [1,79], and analyzing the results from the curves (F${}_{2}$ score vs. FPPI, TPR vs. FPPI and FNR vs. FPPI) over the complete test data set for the separate object classes;
  - By applying the best performance object detection threshold (found from the F${}_{2}$ score vs. FPPI curves for the best performing algorithm for each object class) on the content obtained with each of the TM algorithms, as well as on the SDR content. Then, for the specific best performance object detection threshold for each object class, we measure the F${}_{2}$ score:
    - On the complete test data set;
    - On the split test data set in two categories: “difficult” and “easy” traffic scenes.
∗ Additionally, to compare the complexity of the neural networks, for each of the used CNNs, we measure the number of parameters and the number of multiplication and addition (multiply-accumulate) operations on floating-point numbers (MACs).

#### 4.3.2. Qualitative Analysis

For the qualitative analysis, first, we visually evaluate the results in terms of object detection. Then, we proceed with qualitative analysis on the quality of the reconstructed (demosaicing and tone-mapping) content.

We analyze the quality of the tone-mapping results on several aspects:

- Contrast;
- Color appearance;
- Tone-mapping of strong lights;
- Presence of noise and artifacts.

The quality of demosaicing, in terms of:

- Appearance of disturbing demosaicing artifacts;
- Sharpness of the details;

is analyzed by visual observation on parts of the content with sharp edges and/or repetitive structure.

## 5. Results

In this Section, we present and discuss the results from the quantitative and the qualitative analyses.

### 5.1. Results from the Quantitative Analysis

**Figure 8:**
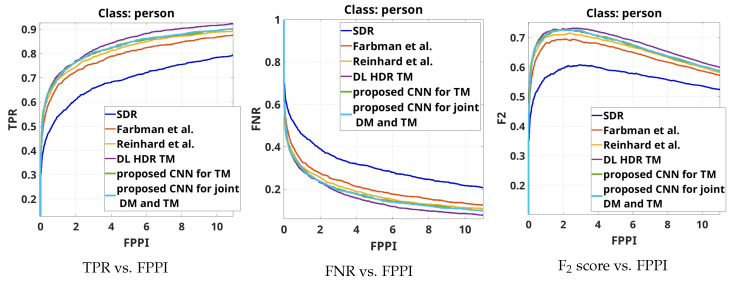
Object detection results for the class “person” on the complete test data set. Note that for the proposed CNNs, the CNN for TM and the CNN for joint DM and TM, the results are very similar to the results for the content obtained with the DL HDR TM [34] algorithm. The slightly better performance of the DL HDR TM algorithm over the proposed algorithms is mainly due to the better sharpness of the objects of interest in the image content. The existence of sharp edges facilitates the object detection of the class “person” in cases of not very distant VRUs and not extremely difficult lighting and contrast conditions. The object class “person” is an object class where the color is not the dominantly important feature for accurate object detection. When compared to the results on the content obtained with the SOTA classical methods for TM, Reinhard et al. [13] and Farbman et al. [28], the proposed CNNs rank high everywhere along the curves. SDR content has the worst object detection performance.

**Figure 9:**
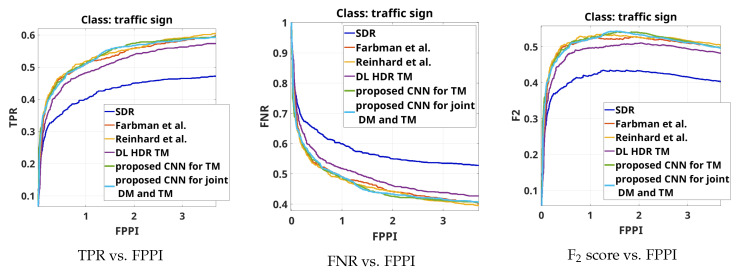
Object detection results for the class “traffic sign” on the complete test data set. Note that the proposed CNNs, the CNN for TM and the CNN for joint DM and TM, achieve the best object detection performance. The results are broadly similar to the results achieved on the content obtained with the TM algorithm proposed by Reinhard et al. [13], and better when compared to the results on the content obtained with Farbman et al. [28] and the DL HDR TM [34] algorithm. The class “traffic sign” is an object class, where both shape and color are dominantly important features for accurate object detection. Conversely, when compared to the other TM algorithms, the content obtained with the DL HDR TM [34] algorithm has the worst object detection results, while SDR content has the worst overall object detection performance.

**Figure 10:**
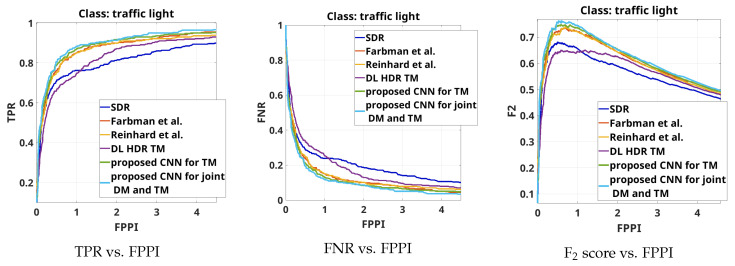
Object detection results for the class “traffic light” on the complete test data set. Note that the content obtained with the proposed CNNs, the CNN for TM and the CNN for joint DM and TM, achieves the best object detection performance. The results from the proposed CNNs are most similar to the results achieved on the content obtained with the TM algorithm proposed by Reinhard et al. [13] and better than results on the content obtained with the TM algorithm proposed by Farbman et al. [28] and the DL HDR TM [34] algorithm. The class “traffic light” is an object class where both shape and color are dominantly important for accurate object detection. Conversely, the content obtained with the DL HDR TM algorithm [34] has the worst object detection performance compared to the content obtained with the other TM algorithms. SDR content has the worst overall object detection performance.

The results from the overall object detection evaluation on the complete test set are shown by the curves TPR vs.FPPI, FNR vs. FPPI and F${}_{2}$ score vs. FPPI for the object class “person” in Figure 8, for the object class “traffic sign”, in Figure 9 and for the object class “colored traffic light”, in Figure 10.

From Figure 8, from the three types of curves, it can be seen that in terms of object detection performance for the class "person", the best-performing algorithm consistently is the SOTA DL HDR TM [34]. The next-best-performing algorithms, with quite similar performance to the DL HDR TM, are the proposed CNN for TM and the proposed CNN for joint DM and TM. In the region of interest (3–4 FPPI), it can be seen that the three best-performing algorithms show almost the same performance. The object detection performance decreases for the classical SOTA algorithms, Farbman et al. [28] and Reinhard et al. [13] and is the poorest for the SDR content. Since the SOTA DL HDR TM increases the contrast around the edges of the objects, it facilitates the object detection of the class “person”, where the clear edges and the shape are the dominant features, while the color fidelity is of less importance. Improvements in the local contrast positively influences the detection of objects that are not too far away (not very small) and the lighting conditions are not extremely difficult. In the cases of distant/small and blurred objects, detection is generally bad for all the algorithms. However, our hypothesis is that in the case of the DL HDR TM algorithm, with the increase in the local contrast, the noise artifacts also become severely pronounced, which negatively affects the detection of small (occluded or distant) objects. Circumstantial evidence toward this hypothesis is provided by the object detection results for traffic signs. In any case, the detection results on the content obtained with the DL HDR TM are only slightly better than those of the proposed CNNs.

From Figure 9, from the three types of curves, it can be seen that the best-performing algorithms with respect to object detection for the object class “traffic sign” in most of the cases, are the proposed CNNs. Similar results are observed from the classical SOTA algorithms, Farbman et al. [28] and Reinhard et al. [13]. The performance is lower for the SOTA DL HDR TM [34], while the SDR content shows poorest performance. In traffic sign detection, beside the shape and the clear edges, the color is also an important feature in the object detection. With the presented object detection results, for the class “traffic sign”, we are justified in saying that in terms of color fidelity in tone-mapping, the proposed CNNs produce high-quality image content and show good performance.

Similar conclusions can be obtained if the object detection results presented in Figure 10 are analyzed. It can also be seen that, when it comes to accurately tone-mapping traffic lights without an increase in artifacts in their vicinity, the DL HDR TM algorithm [34] in parts shows poorer performance than the SDR content.

**Figure 11:**
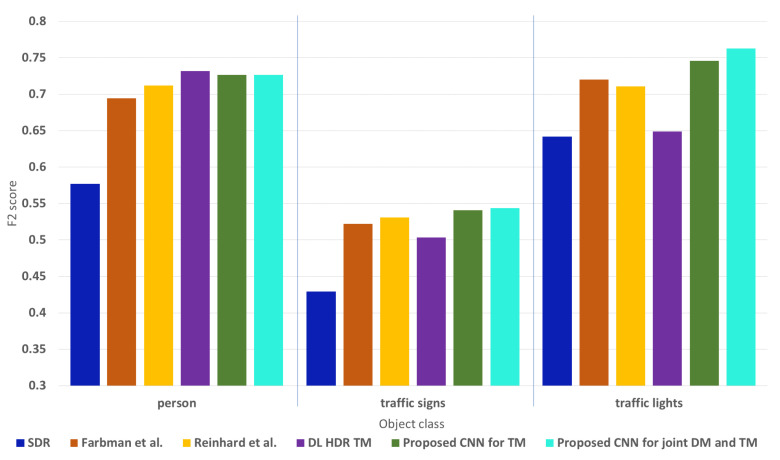
Object detection results ($F_{2}$ score) for the classes “person”, “traffic sign” and “colored traffic light”. The presented $F_{2}$ score results are obtained on the content from the algorithms in the comparison for the same working point/object detection threshold of the object detector in each object class. The working points (the object detection thresholds), for each object class, are selected from the best F2-score value (from the results presented in Figure 8, Figure 9 and Figure 10) of the best performing algorithm for each object class. The selected thresholds, per object class are then applied in the object detection on the content obtained with all the algorithms. Note that the content obtained with the proposed CNNs, the CNN for TM and the CNN for joint DM and TM, achieves best results for the classes “traffic sign” and “colored traffic light” and similar results to those of the best-performing algorithm (the DL HDR TM algorithm [34]) for the class “person”.

We support the obtained conclusions for the overall object detection analysis with the F${}_{2}$ score results presented in Figure 11. Here, the F${}_{2}$ score is calculated for every object class over the complete test data set. The working point, i.e., the object detection threshold, is selected from the best F${}_{2}$ score value of the best-performing algorithm in every object class. This object detection threshold is then applied to the content obtained with the other algorithms from the comparison and also to the SDR content. It can be seen that consistently, the content obtained with the proposed CNNs shows best or second-best object detection performance (although the difference in performance in those cases is negligible) for the evaluated object classes.

**Figure 12:**
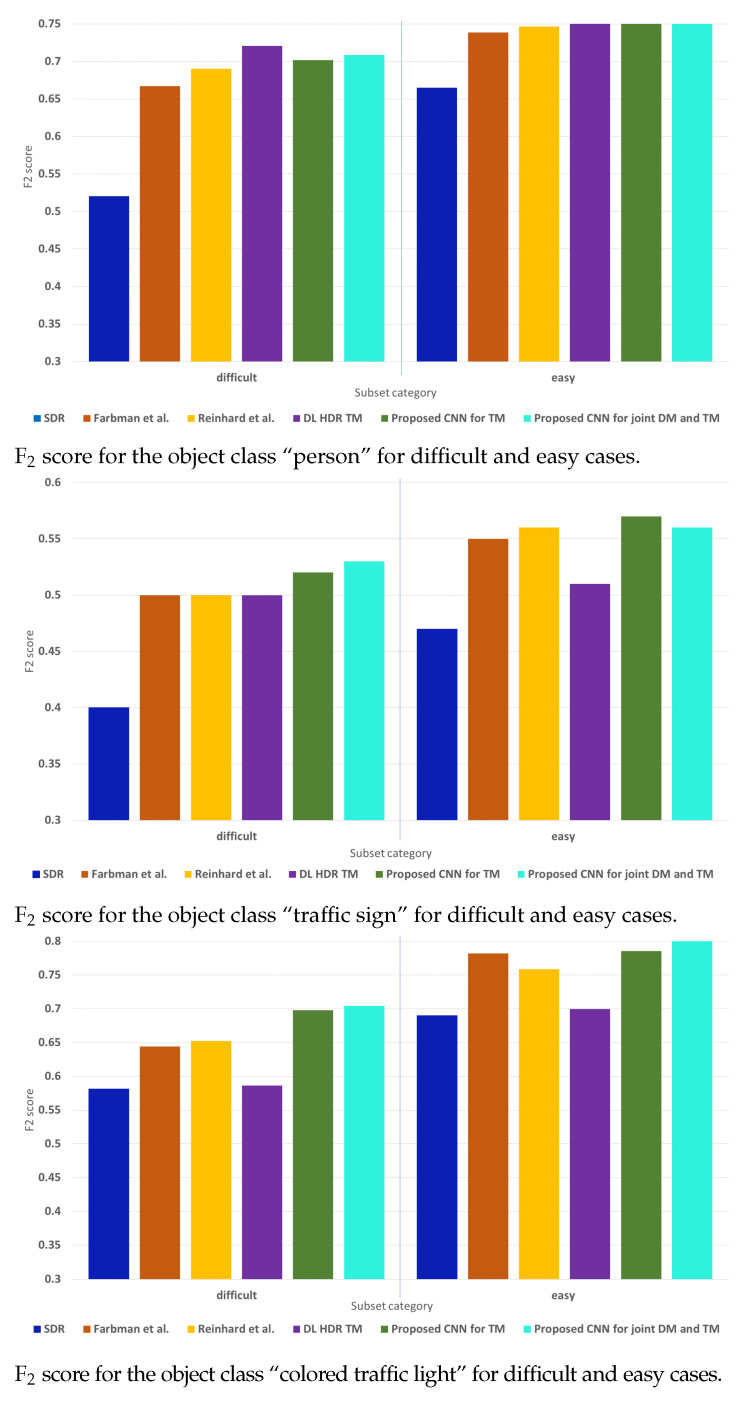
Object detection results ($F_{2}$ score) on the split test data set (difficult and easy cases), for each of the evaluated object classes. Note that the content obtained with the proposed CNNs, the CNN for TM and the CNN for joint DM and TM, achieves best results in most of the cases in both subsets (“difficult” and “easy”) of the test data set. Slightly better performance is only achieved by the DL HDR TM algorithm [34] for the subset of “difficult scenes” for the object class “person”. This is not the case for the other two object classes, “traffic sign” and “colored traffic light”. Furthermore, note that the overall performance is better for the subset of “easy” scenes compared to the subset of “difficult” scenes. The difference in performance between the TM algorithms is the highest and in favor of the proposed CNNs in the subset of the “difficult” traffic scenes.

The results from the separate evaluation on the difficult and the easy scenes from the test data set are shown in Figure 12. The working points, i.e., the object detection thresholds, retain the same selected values as for the results presented in Figure 11. For the object class “person”, the performance on the easy scenes for all the algorithms from the comparison is similar, while on the difficult scenes, the best-performing algorithm is the DL HDR TM [34]. For the difficult scenes, the content obtained with the DL HDR TM has only slightly better object detection results than the results on the content from the proposed CNNs. When compared to the classical SOTA TM algorithms, the proposed CNNs are better. Overall, the performance is better on the easy scenes than on the difficult scenes. For the object class “traffic sign”, the best-performing algorithm in the difficult cases is the proposed CNN for joint DM and TM, while for the easy cases, it is the proposed CNN for TM. For the difficult cases, the algorithms from the comparison show similar performance to each other and perform worse than the proposed CNNs. For the easy cases, worst in performance among the algorithms from the comparison is the DL HDR TM [34], due to the infidelity in color tone-mapping. For the object class “colored traffic light”, the conclusions are similar, with the difference being that the best-performing algorithm is the proposed CNN for joint DM and TM for both the difficult and easy cases. When compared with the proposed CNN for TM, the difference in performance of the proposed CNNs is negligible. Overall, the performance is better on the easy traffic scenes than on the difficult traffic scenes. Furthermore, for all of the evaluated object classes, in most of the cases, of the classical SOTA algorithms, the algorithm of Reinhard et al. [13] shows better performance than the algorithm of Farbman et al. [28], and the SDR content consistently shows the worst performance.

As part of the quantitative analysis, in order to test our second hypothesis, we also calculated the computational cost (number of parameters and multiply-and-accumulate units (MACs)) of the proposed CNNs. The results are presented in Table 1. In terms of number of trainable parameters, the results show that the proposed CNNs do not differ much and have similar numbers of parameters. The proposed CNN for joint DM and TM has a slightly larger architecture because of the additional layers. However, it is not drastically larger than the proposed CNN for TM; on the contrary, they are similar in size. Moreover, the proposed CNN for joint DM and TM performs the task of the proposed CNN for TM, additionally with demosaicing, in an optimal, joint manner. A similar conclusion can be obtained if the MACs results are compared.

With the presented results from the quantitative analysis, we justify the use of the proposed CNN for TM and the proposed CNN for joint DM and TM in tone-mapping (and demosaicing) of difficult, as well as easy, HDR traffic scenes, with the purpose of improving the detectability of traffic-related objects. Our hypothesis is that the object detection performance is improved due to the better contrast, the noise suppression, the absence of disturbing artifacts and the high color fidelity in the content reconstructed with the proposed CNNs. We further confirm this hypothesis with the results from the qualitative analysis.

Additionally, with the quantitative analysis, we show that the two proposed CNNs, at almost the same computational cost, achieve very similar object detection performance on the reconstructed content.

### 5.2. Results from the Qualitative Analysis

Here, we first qualitatively analyze and discuss the object detection results. We then proceed with the qualitative analysis of the reconstructed content with the algorithms from the comparison and the proposed CNNs.

#### 5.2.1. Qualitative Analysis of the Object Detection Results

The results from the quantitative analysis are supported with visual examples from the tone-mapped content and the detections shown as rectangles (green rectangle: True Positive detection, red rectangle: False Negative, i.e., missed detection, and orange rectangle: False Positive). With these examples, we visually inspect the reasons for the good or bad object detection performance on the content obtained with the pipelines of:

- The sequentially combined, SOTA demosaicing and TM algorithms;
- The sequentially combined, SOTA demosaicing and the proposed CNN for TM;
- The proposed CNN for joint DM and TM.

**Figure 13:**
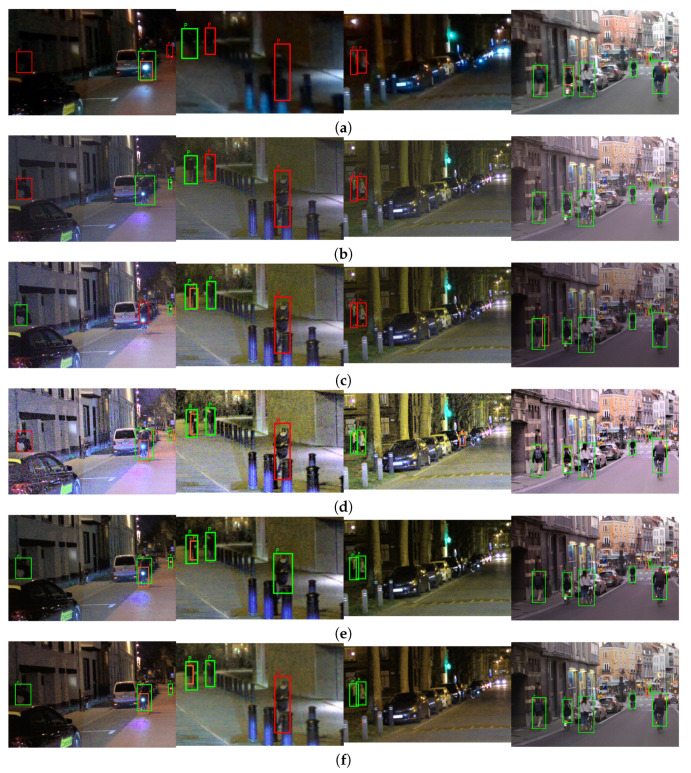
Visually presented results from object detection performance for the class “person” on selected examples from the test data set. Image order: left-to-right. The images from the first to the third image in every row are examples from the “difficult” traffic scenes. The fourth image is an example from the “easy” traffic scenes. A red rectangle denotes a missed detection (FN), an orange rectangle denotes a False Positives (FP) and a green rectangle denotes a True Positives (TP).(**a**) Detection results on the SDR content; (**b**) Detection results on the content obtained with the pipeline: Menon et al. [56] and Farbman et al. [28]; (**c**) Detection results on the content obtained with the pipeline: Menon et al. [56] and Reinhard et al. [13]; (**d**) Detection results on the content obtained with the pipeline: Menon et al. [56] and the DL HDR TM algorithm [34]; (**e**) Detection results on the content obtained with the pipeline: Menon et al. [56] and the proposed CNN for TM; (**f**) Detection results on the content obtained with the proposed CNN for joint DM and TM. Note that in the extremely “difficult” scenes (strong lights or low contrast and low-light conditions), in most of the cases, the proposed CNNs achieve object detection results with fewest missed detections. In the “easy” case, the object detection results between all algorithms are almost same. The object detection results are worst for the SDR content.

**Figure 14:**
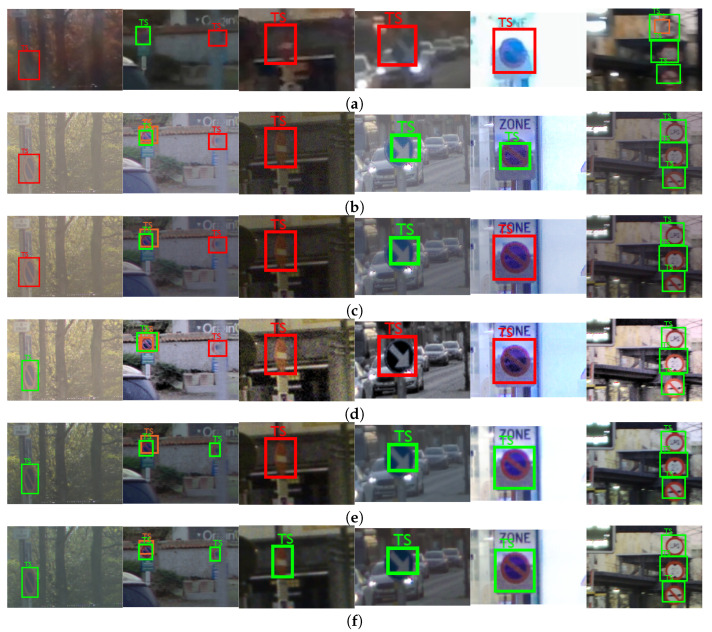
Visually presented results from object detection performance for the class “traffic sign” on selected examples from the test data set. Image order: left-to-right. The images from the first to the fifth image in every row are examples from the “difficult” traffic scenes. The sixth image is an example from the “easy” traffic scenes.A red rectangle denotes a missed detection, an orange rectangle denotes an FP and a green rectangle denotes a TP. (**a**) Detection results (class “traffic sign”) on the SDR content; (**b**) Detection results (class “traffic sign”) on the content obtained with the pipeline: Menon et al. [56] and Farbman et al. [28]; (**c**) Detection results (class “traffic sign”) on the content obtained with the pipeline: Menon et al. [56] and Reinhard et al. [13]; (**d**) Detection results (class “traffic sign”) on the content obtained with the pipeline: Menon et al. [56] and the DL HDR TM algorithm [34]; (**e**) Detection results (class “traffic sign”) on the content obtained with the pipeline: Menon et al. [56] and the proposed CNN for TM; (**f**) Detection results (class “traffic sign”) on the content obtained with the proposed CNN for joint DM and TM. Note that in the “difficult” scenes, in most of the cases, the proposed CNNs achieve object detection results with fewest missed detections. The best results are achieved by the proposed CNN for joint DM and TM. On the presented “easy” scene, the object detection results of all algorithms are the same. The object detection results are worst for the SDR content.

**Figure 15:**
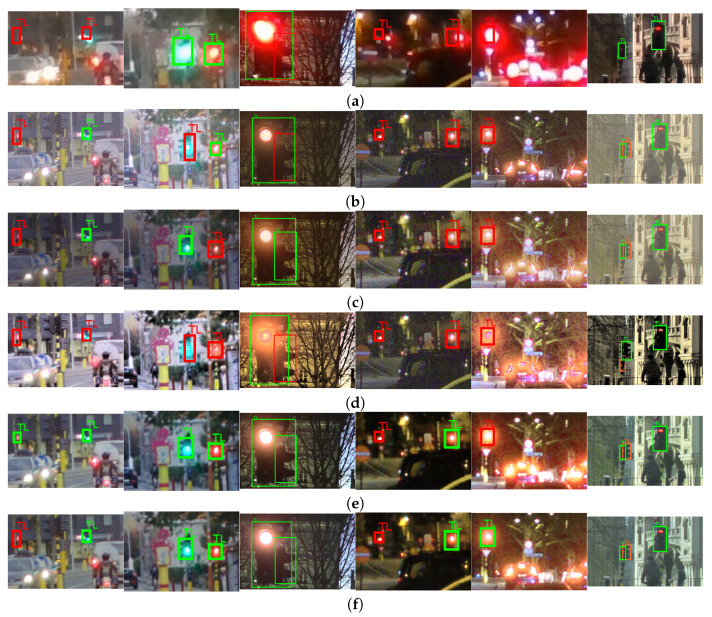
Visually presented results from object detection performance for the class “colored traffic light” on selected examples from the test data set. Image order: left-to-right. The images from the first to the fifth image in every row are examples from the “difficult” traffic scenes. The sixth image is an example from the “easy” traffic scenes. A red rectangle denotes a missed detection, an orange rectangle denotes an FP and a green rectangle denotes a TP. (**a**) Detection results on the SDR content; (**b**) Detection results on the content obtained with the pipeline: Menon et al. [56] and Farbman et al. [28]; (**c**) Detection results on the content obtained with the pipeline: Menon et al. [56] and Reinhard et al. [13]; (**d**) Detection results on the content obtained with the pipeline: Menon et al. [56] and the DL HDR TM algorithm [34]; (**e**) Detection results on the content obtained with the pipeline: Menon et al. [56] and the proposed CNN for TM; (**f**) Detection results on the content obtained with the proposed CNN for joint DM and TM. Note that in the “difficult” scenes, in most of the cases, the proposed CNNs achieve object detection results with the fewest missed detections. The best results are achieved by the proposed CNN for joint DM and TM. On the “easy” case, the object detection results between all algorithms are almost same. The object detection results are worst for the SDR content and the content obtained with the pipeline: Menon et al. [56] and the DL HDR TM algorithm [34].

The visual examples, difficult and easy traffic scenes for the object class “person” are shown in Figure 13, for the object class “traffic sign”, are shown in Figure 14, and for the object class “colored traffic light”, are shown in Figure 15.

The difficult scenes shown in Figure 13 consider cases of very strong lights in the vicinity of the relevant objects, poor contrast and occluded pedestrians. It can be seen that the objects from the class “person” are extremely hard to distinguish in the SDR content. Most of the objects are detected in the content obtained with the proposed CNNs and the DL HDR TM [34]. The difference in the visual appearance between the content obtained with the proposed CNNs and the DL HDR TM [34] algorithm is quite noticeable. The scenes tone-mapped with the DL HDR TM [34] algorithm are brighter, the sharpness of the edges is greater and the noise is severe and with granular structure. The noise appearance has a negative effect on the detection, which can be observed from the content obtained with the algorithms from the comparison. Specifically, objects that are already hard for detection, such as the occluded person in the dark left part of the scene (in the first image from left-to-right), the partially occluded person on the bicycle with strong front lights (in the first image from left-to-right), are detected only in the content obtained with the proposed CNNs. Additionally, due to the improved contrast (see the examples presented from the first to the third image in the left-to-right order) after tone-mapping with the proposed CNNs, most of the objects become detectable, which is not the case with the other TM algorithms. Aside from the severe noise presence in the algorithms from the comparison, we can say that the visual appearance of the content tone-mapped with the proposed CNNs is most similar to the visual appearance of the content tone-mapped with the classical SOTA algorithms [13,28]. Positive aspects of the proposed CNNs are the improvements in the contrast and the amount of noise. For the easy case of traffic scenes (fourth image from left-to-right), the objects are detected with all of the algorithms included in the comparison, as well as in the SDR content.

The difficult scenes shown in Figure 14 consider cases of sun glare (first image from left-to-right), strong lights (fourth and fifth image from left-to-right), poor contrast (third and fourth image from left-to-right), motion of the camera (second image from left-to-right) and occlusion (second image from left-to-right). It can be seen that most of the objects from the class “traffic sign” are detected in the content tone-mapped with the proposed CNNs, specifically the proposed CNN for joint DM and TM. The fourth image from left-to-right is a good example of a difficult case in terms of accurate color tone-mapping. Some ROI parts in the content obtained with the DL HDR TM [34] algorithm lack color saturation, which makes the traffic signs less detectable. The behavior is similar for the case from the fifth image from left-to-right. On the third image example from left-to-right, it can be seen that the noise is very pronounced and the contrast is very poor, and these aspects severely affect the object detection with the algorithms from the comparison. The traffic sign of interest from the third image is only detected in the proposed CNN for joint DM and TM. The noise and the poor contrast are the main reasons for the missed detections from the “difficult cases” in the content obtained with the classical SOTA tone-mapping algorithms: [13,28]. In the presented visual example, for the easy case (sixth image from left-to-right), the objects are detected in the content obtained with all of the algorithms included in the comparison, as well as in the SDR content.

The difficult scenes in Figure 15 consider cases of strong lights in the vicinity (from the first to the fifth image from left-to-right) of the traffic lights and poor contrast in the local regions of the traffic lights (from the first to the fourth image from left-to-right). Most of the objects are detected in the content obtained with the proposed CNNs. For the scene on the fifth image from left-to-right, which is an extremely difficult case, there are no traffic lights detected in any of the content obtained with the SOTA algorithms from the comparison. On the contrary, one traffic light is detected only in the content obtained with the proposed CNN for joint DM and TM. This points to the advantage of the proposed CNN for joint TM and DM to be used in demosaicing and tone-mapping of extremely difficult cases. The situation is similar for the scene from the fourth image. Traffic lights are only detected in the content obtained with the proposed CNNs. In this specific scene, the noise presence is severe in the content obtained with the DL HDR TM [34] algorithm and also quite noticeable, although to a lesser extent, in the content obtained with the classical SOTA algorithms [13,28]. Conversely, the noise is highly suppressed and the contrast is improved in the content obtained with the proposed CNNs, hence, more of the traffic lights are detected. For the same reason, some of the traffic lights (second image from left-to-right) are detected in the SDR content, too. For some of the scenes, where some of the traffic lights are very strong, the DL HDR TM [34] algorithm fails to accurately tone-map the local region of the light source and creates a large halo artifact with severe noise artifacts in the light source surroundings. The “halo” artifacts around the light sources are bad because they change the local contrast, hence, the surrounding structures become less visible, negatively affecting the object detection performance. These “halo” artifacts with additional noise artifacts, are especially noticeable in the scenes from the second image, the third image and the fifth image from left-to-right in the content obtained with the SOTA DL HDR TM. Large “halo” artifacts do not appear after tone-mapping when the proposed CNNs and the other TM algorithms from the comparison are applied. Another positive aspect is that, in the cases where the contrast is generally poor (scenes from the first to the third image from left-to-right), in the surroundings of the traffic lights, the best results in terms of object detection are achieved for the content obtained with the proposed CNNs. For the easy scenes (the sixth image from left-to-right), the colored traffic lights are detected in the content obtained with all of the algorithms included in the comparison, as well as in the SDR content.

#### 5.2.2. Qualitative Analysis of the Tone-Mapping and Demosaicing Results

Here, for the qualitative analysis, we visually present the results of tone-mapping, as well as demosaicing, and discuss the different aspects of the obtained visual quality in tone-mapping and demosaicing, with the proposed CNNs and the SOTA algorithms from the comparison.

**Figure 16:**
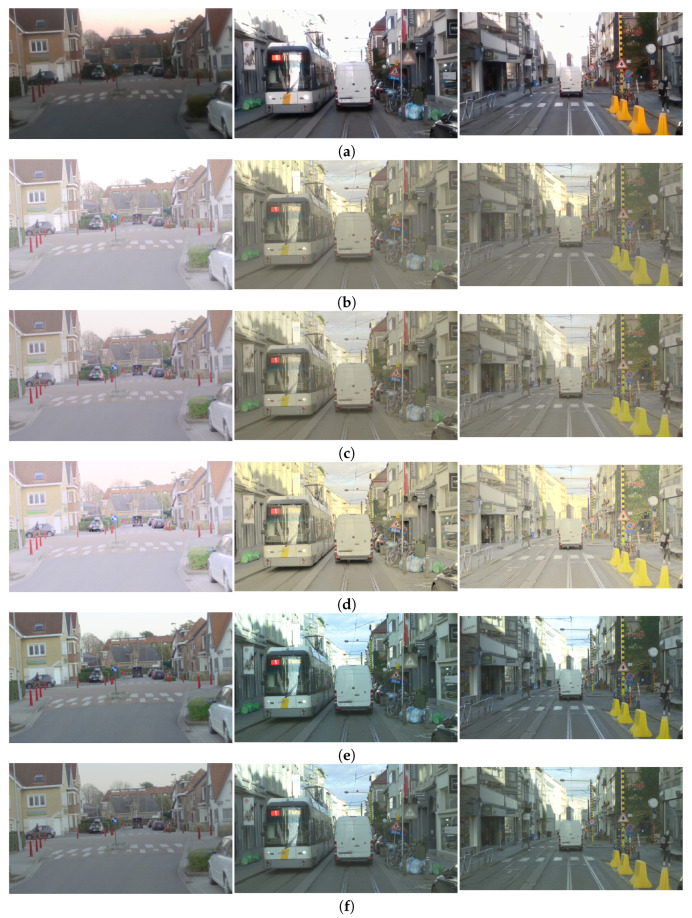
Visual results from the qualitative analysis of the performance quality concerning contrast. Image order: left-to-right. (**a**) SDR content; (**b**) Content obtained with the pipeline: Menon et al. [56] and Farbman et al. [28]; (**c**) Content obtained with the pipeline: Menon et al. [56] and Reinhard et al. [13]; (**d**) Content obtained with the pipeline: Menon et al. [56] and the DL HDR TM algorithm [34]; (**e**) Content obtained with the pipeline: Menon et al. [56] and the proposed CNN for TM; (**f**) Content obtained with the proposed CNN for joint DM and TM.

**Figure 17:**
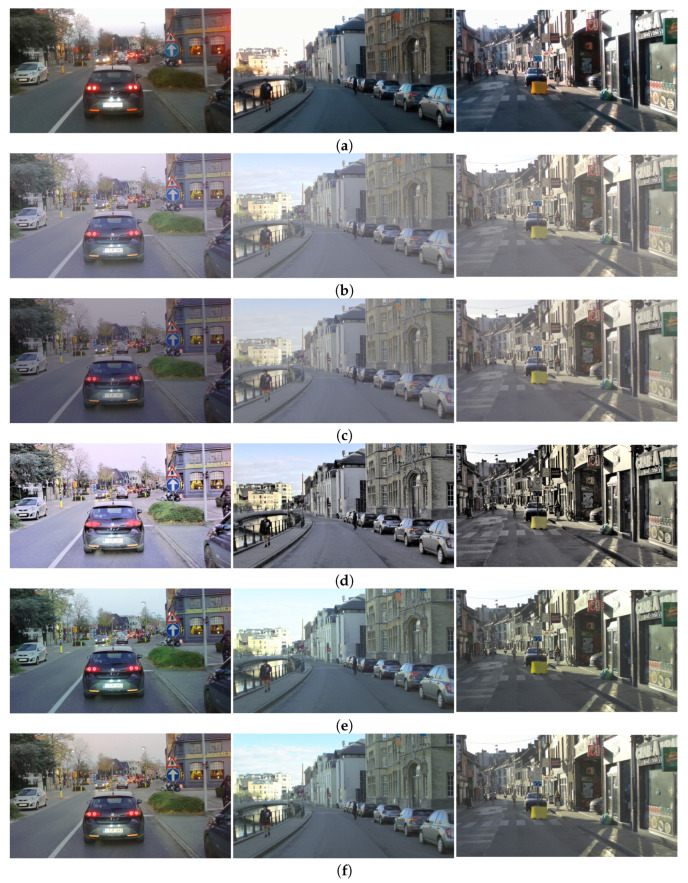
Visual results from the qualitative analysis of the performance quality concerning color appearance. Image order: left-to-right. (**a**) SDR content; (**b**) Content obtained with the pipeline: Menon et al. [56] and Farbman et al. [28]; (**c**) Content obtained with the pipeline: Menon et al. [56] and Reinhard et al. [13]; (**d**) Content obtained with the pipeline: Menon et al. [56] and the DL HDR TM algorithm [34]; (**e**) Content obtained with the pipeline: Menon et al. [56] and the proposed CNN for TM; (**f**) Content obtained with the proposed CNN for joint DM and TM.

**Figure 18:**
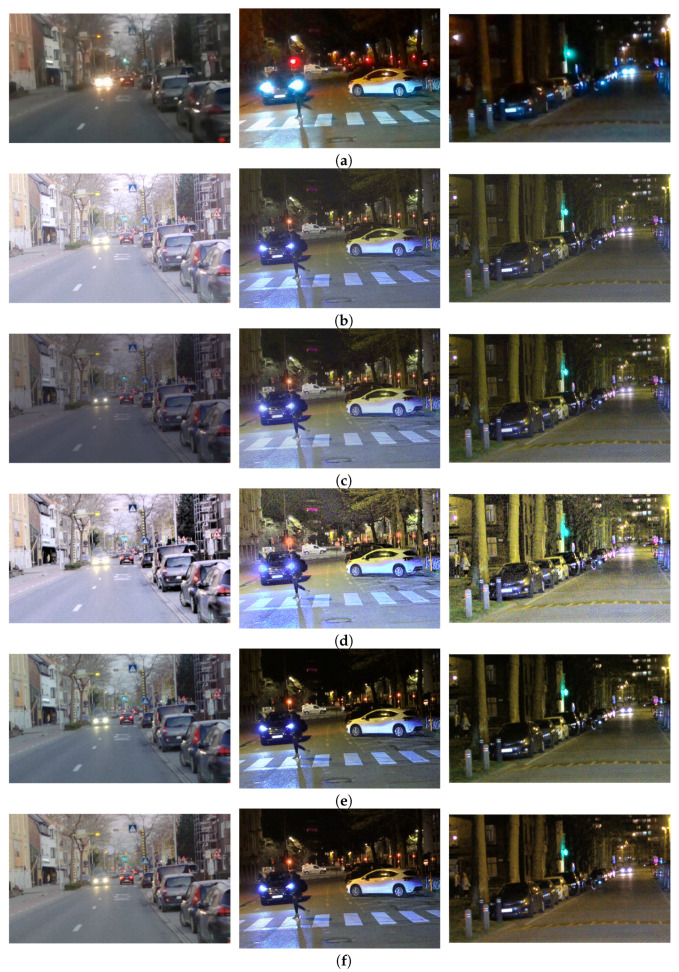
Visual results from the qualitative analysis of the performance quality concerning tone-mapping of strong lights. (**a**) SDR content; (**b**) Content obtained with the pipeline: Menon et al. [56] and Farbman et al. [28]; (**c**) Content obtained with the pipeline: Menon et al. [56] and Reinhard et al. [13]; (**d**) Content obtained with the pipeline: Menon et al. [56] and the DL HDR TM algorithm [34]; (**e**) Content obtained with the pipeline: Menon et al. [56] and the proposed CNN for TM; (**f**) Content obtained with the proposed CNN for joint DM and TM.

**Figure 19:**
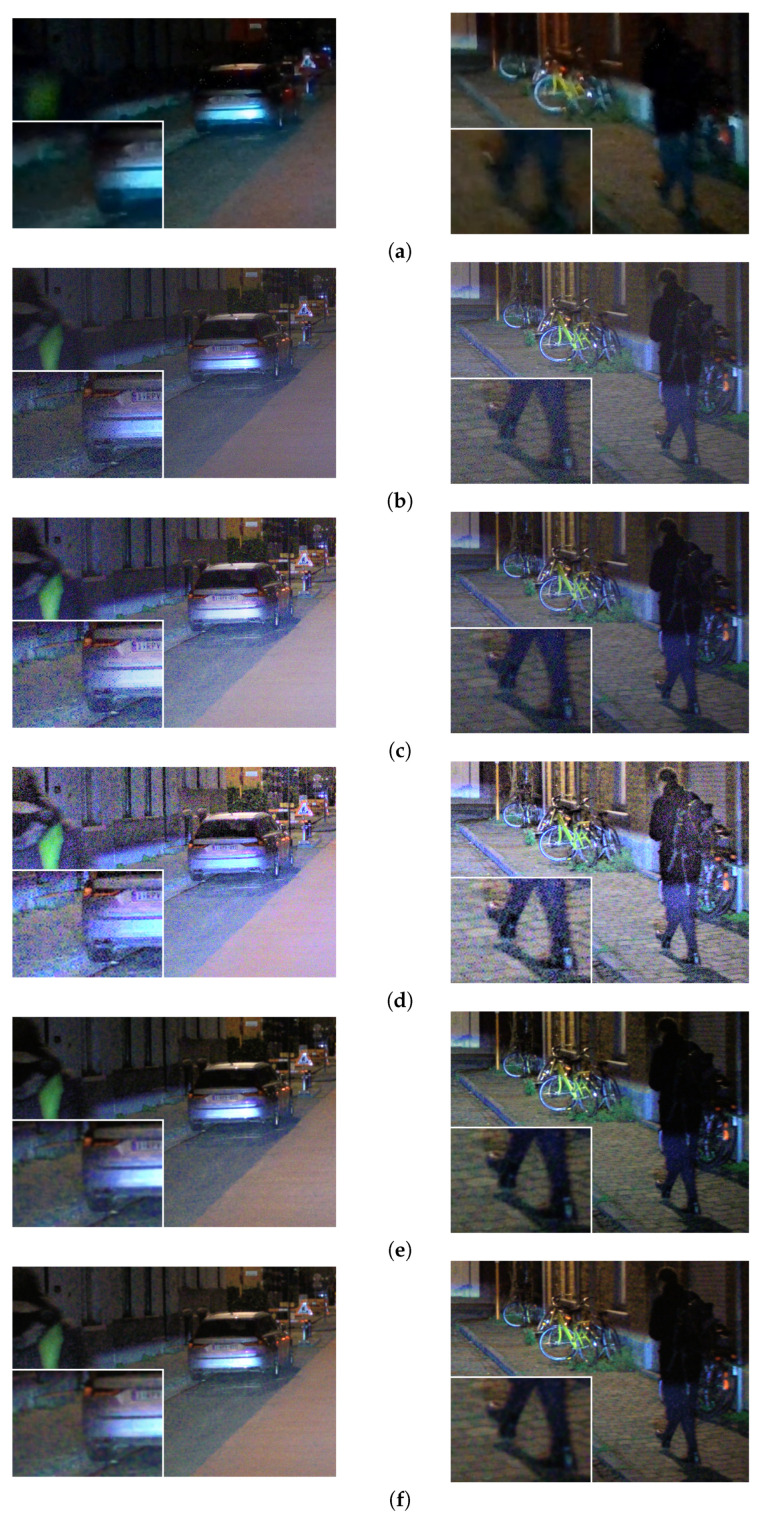
Qualitative results (presented with zoomed image crops) of the performance quality concerning the presence of noise. (**a**) SDR content; (**b**) Content obtained with the pipeline: Menon et al. [56] and Farbman et al. [28]; (**c**) Content obtained with the pipeline: Menon et al. [56] and Reinhard et al. [13]; (**d**) Content obtained with the pipeline: Menon et al. [56] and the DL HDR TM algorithm [34]; (**e**) Content obtained with the pipeline: Menon et al. [56] and the proposed CNN for TM; (**f**) Content obtained with the proposed CNN for joint DM and TM.

**Figure 20:**
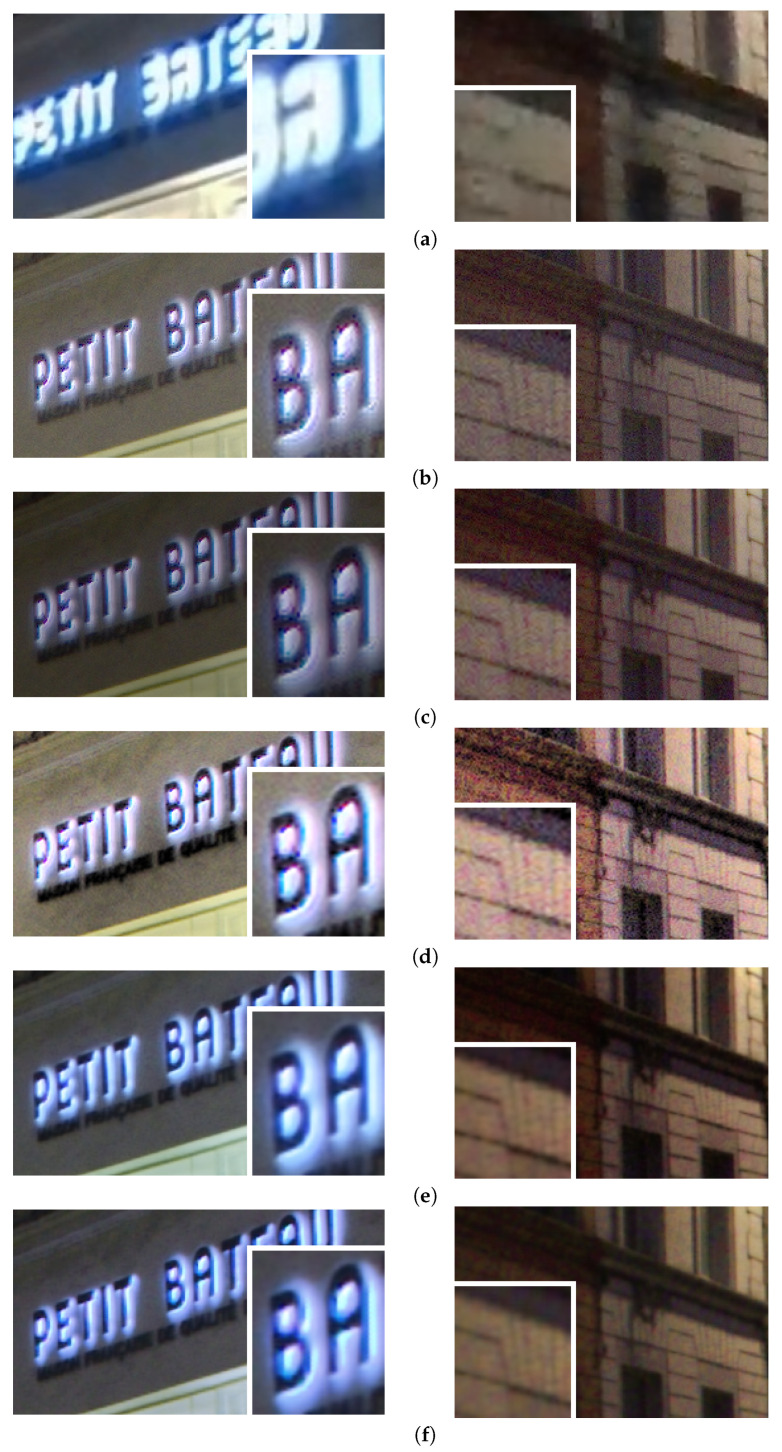
Visual results (presented with zoomed image crops) from the qualitative analysis of the performance quality concerning demosaicing. (**a**) SDR content; (**b**) Content obtained with the pipeline: Menon et al. [56] and Farbman et al. [28]; (**c**) Content obtained with the pipeline: Menon et al. [56] and Reinhard et al. [13]; (**d**) Content obtained with the pipeline: Menon et al. [56] and the DL HDR TM algorithm [34]; (**e**) Content obtained with the pipeline: Menon et al. [56] and the proposed CNN for TM; (**f**) Content obtained with the proposed CNN for joint DM and TM.

For the results presented in Figure 16, we discuss the contrast; for the results presented in Figure 17, we discuss the tone-mapping as far as the color appearance is concerned; for the results presented in Figure 18, we discuss the tone-mapping of strong lights and in the vicinity of strong lights; for the results presented in Figure 19, we discuss the noise appearance in the differently tone-mapped content; and for the results presented in Figure 20, we discuss the demosaicing quality.

With visual observation of the examples presented in Figure 16, it can be seen that the content with pleasant visual appearance for the HVS and with high global and local contrast is obtained with the proposed CNNs. This can not be stated for the content obtained with the other algorithms from the comparison. For the content tone-mapped with the DL HDR TM [34], there are cases where the overall contrast, as it is in the presented examples of bright daytime scenes, is lower than the contrast in the content obtained with the proposed CNNs. Furthermore, very often, the global contrast in the tone-mapped content with the DL HDR TM algorithm may be very high and the edges of the objects may have strong sharpness, to a level that the tone-mapped content starts to resemble “cartoon-like content”. Such cases can be observed in Figure 17, the second and the third image from left-to-right. The situation is similar for the content obtained with the classical SOTA tone-mapping algorithms: Reinhard et al. [13] and Farbman et al. [28].

From the examples presented in Figure 17, it can be seen that the colors are more saturated, more apparent and more vivid in the content obtained with the proposed CNNs. It can also be seen that the content obtained with the proposed CNNs resembles the SDR content in terms of color appearance, which means that the proposed CNNs do not perform incorrect color tone-mapping. This is a very important aspect in the detection of traffic signs and traffic lights. The colors are less saturated in the content tone-mapped with the DL HDR TM [34] algorithm, and to a lower extent, less saturated in the content tone-mapped with the classical SOTA algorithms, Reinhard et al. [13] and Farbman et al. [28]. Additionally, for the content tone-mapped with the DL HDR TM [34] algorithm, there are cases with wrong color tone-mapping, especially if the example from the first image from left-to-right, is observed. In this image example, the red back-lights of the car are uniformly tone-mapped in the content obtained with the DL HDR TM algorithm. Conversely, there is a visible distinction between the color-saturated and the luminance-saturated regions in the content obtained with the other algorithms, implying that, indeed, the color tone-mapping for this specific example is wrong when the DL HDR TM [34] is applied.

From the examples presented in Figure 18, it can be seen that the strong lights are tone-mapped without large halo artifacts around the light source (and hence, the light source is well localized) with the proposed CNNs and the algorithm by Reinhard et al. [13]. The clipping and the halo artifacts become slightly more pronounced in the content tone-mapped with the algorithm by Farbman et al. [28]. The halo artifacts become severe, with surrounding noise artifacts, in the content obtained with the DL HDR TM [34] algorithm.

In Section 3.5.7, we elaborated that the photon noise (noise with a Poisson distribution), initially present in the CFA mosaiced image, after demosaicing, becomes severe, with noise artifacts and with complex structure. For that reason, the same noise artifacts are likely to appear in the tone-mapped content as well. From the examples presented in Figure 19, it can be seen that the noise is most severe (most pronounced and with granular structure) in the content tone-mapped with the DL HDR TM [34] algorithm. It is present and noticeable, although not so disturbing, in the content tone-mapped with the other classical SOTA algorithms, Reinhard et al. [13] and Farbman et al. [28]. Fortunately, it is suppressed to a large extent in the content obtained with the proposed CNNs (with slightly better suppression in the content obtained with the proposed CNN for joint DM and TM).

From the examples presented in Figure 20, it can be seen that there are no very disturbing demosaicing artifacts; however, they do exist and interfere with the noise structure. The HDR content prior to being processed with the tone-mapping algorithms has been demosaiced with the algorithm of Menon et al. [56]. Therefore, the artifacts in the content obtained with the sequential pipelines of demosaicing and tone-mapping algorithms occur in the same place, and are more or less pronounced. The intensity to which they are pronounced depends on how the tone-mapping algorithms deal with that problem. A good example with the presence of demosaicing artifacts is given in the first image from left-to-right (note the wrong color pixels around the text in the content obtained with the sequential pipelines). The classical tone-mapping algorithms, Reinhard et al. [13] and Farbman et al. [28], do not perform denoising, so the artifacts remain, only their magnitude as pixels with wrong color value becomes increased. These artifacts are severely pronounced in the content tone-mapped with the DL HDR TM [34] algorithm. This is due to the fact that the DL HDR TM, in an attempt to maximize the local contrast, makes the artifacts more apparent. Fortunately, the proposed CNN for TM, since it has been trained on noisy data and data with artifacts from demosaicing, in most of the cases, suppresses these types of artifacts and tone-maps the content according to the local surroundings and the local spatial activity. From this aspect, we point to the advantage of our proposed CNN for TM to suppress noise and demosaicing artifacts in the tone-mapped content. The situation is similar, with slight worsening, for the content obtained with the proposed CNN for joint DM and TM. The effect of slight blurring, however, with no additional artifacts, can be seen in the second image from left-to-right when the proposed CNNs are applied. The suppression of artifacts and noise for the proposed CNN for joint DM and TM comes at a cost of not having a very high sharpness of the edges in the reconstructed content. However, as long as the content is not noticeably blurred, high global and local contrast is ensured, and the objects are well detectable (i.e., object detection is not negatively affected), while still producing visually pleasant content for the HVS, this is an acceptable compromise made with the proposed CNNs.

With the results from the qualitative analysis, we are again justified in claiming that the proposed CNNs for tone-mapping and demosaicing succeed in increasing the detectability of objects and in that regard, are useful in both ADS and AVS. We show that the proposed CNNs outperform the SOTA demosaicing and tone-mapping algorithms:

- In consistently obtaining overall high global and local contrast;
- In obtaining high fidelity in color tone-mapping especially for traffic signs and traffic lights;
- In performing localized tone-mapping of the strong lights (without producing “halo” artifacts in the surroundings);
- In performing noise suppression;
- In obtaining robustness to demosaicing and noise artifacts.

We also show that the proposed CNNs obtain very similar qualitative results. The CNN for joint DM and TM succeeds in achieving almost the same quality as the proposed CNN for TM without noticeable visual differences, with no additional artifacts, with almost the same computational cost and most importantly, by performing both tasks of demosaicing and tone-mapping in an optimal, joint manner.

## 6. Conclusions and Future Work

For use in automotive driving systems, we propose two neural networks, one that performs tone-mapping on demosaiced HDR content and a second that performs joint demosaicing and tone-mapping on raw HDR content. The main idea is to overcome the limitations of the SDR content and the limitations arising from the SOTA object detection algorithms designed to work on 8-bit content, by using HDR content and its proper tone-mapping. We focus on increasing the detectability of the VRUs and traffic-related objects important for road safety in various challenging illumination and contrast conditions. We achieve this by increasing the contrast, maintaining the color appearance, suppressing the noise and avoiding artifacts introduction in both difficult and easy traffic scenes. We evaluate the content produced by the proposed CNNs, with regards to ADS object detection accuracy and visual qualitative analysis. We compare the performance of the proposed CNNs to that of the sequential pipelines of applied SOTA demosaicing and tone-mapping algorithms, as well as to the SDR content. Additionally, we compare the computational cost between the proposed CNNs.

There are two main contributions of this research:

- Incorporating demosaicing in a neural network devised for tone-mapping. Based on the proposed CNN model for TM, we devise a CNN that performs joint demosaicing and tone-mapping of HDR content with noise suppression and good robustness on artifacts occurrence. The proposed CNN for joint DM and TM also has light-weight architecture and only slightly higher computational cost than the proposed CNN for TM.
- Extensive evaluation with respect to ADS object detection accuracy of reconstructed (demosaiced and tone-mapped) content obtained with SOTA demosaicing and tone-mapping algorithms and the proposed CNNs. There is also a discussion and analysis on the quality of the reconstructed content by addressing the main aspects of high quality tone-mapping and demosaicing.

With the results from both the quantitative analysis and the qualitative analysis, we confirm our two research hypotheses:

- The content obtained with the proposed CNN models shows similar or distinctively better ADS object detection performance compared to the content obtained with the SOTA tone-mapping and demosaicing algorithms and also compared to the SDR content. Specifically, with the proposed CNNs, we succeed in improving the detectability of traffic-related objects and pedestrians over two existing fundamental cases, that of sequential demosaicing and tone-mapping of HDR data and only using the SDR content.
- With the obtained similar computational cost between the proposed CNNs and the very similar results from the quantitative and the qualitative analysis, we confirm our second hypothesis.

With the results from the quantitative and the qualitative analysis, we justify the use of the two proposed CNNs in automotive driving and vision systems.

Our future research will include improving the sharpness around the edges in the tone-mapped and demosaiced content without the introduction of artifacts and with satisfying noise suppression; and reconstruction of HDR scenes of diverse weather conditions, including fog, rain (light or heavy rain), snow etc., with the purpose of increasing detectability and facilitating ADS object detection in such scenes, too.

## Figures and Tables

**Table 1:** Computational cost and number of parameters of the proposed neural networks.

Neural Network	Number of Parameters	MACs in G (Input Image Size: 1920 × 1080 × 3)
Proposed CNN for TM	340,227	32,535 G
Proposed CNN for joint DM and TM	340,587	32,668 G

## Data Availability

The data presented in study study are available on request from the corresponding authors. The data are not publicly available due to privacy restrictions.

## Abbreviations

The following abbreviations are used in this manuscript:

HDR	High dynamic range
SDR	Standard dynamic range
ADS	Automated driving systems
AVS	Automotive vision systems
CNN	Convolutional neural network
TM	tone-mapping
CNN for joint DM and TM	Convolutional neural network for joint demosaicing and tone-mapping
CNN for TM	Convolutional neural network for tone-mapping
DL HDR TM	Deep-learning high dynamic range tone-mapping
LDR	Low dynamic range
SOTA	state-of-the-art
CFA	Color filter array
AD	Analog to digital
MACs	Multiply-accumulate operations
PSNR	Peak Signal-to-Noise Ratio
SSIM	Structural Similarity Index
HVS	Human visual system
FP	False Positives
TP	True Positives
TN	True Negatives
FPPI	False Positives per Image

## References

[1] Redmon J., Farhadi A. (2018). YOLOv3: An Incremental Improvement. arXiv.

[2] Shopovska I., Stojkovic A., Aelterman J., Van Hamme D., Philips W. (2023). High-Dynamic-Range Tone Mapping in Intelligent Automotive Systems. Sensors.

[3] Eilertsen G., Mantiuk R.K., Unger J. (2017). A comparative review of tone-mapping algorithms for high dynamic range video. Comput. Graph. Forum.

[4] Eilertsen G. (2018). The High Dynamic Range Imaging Pipeline.

[5] Banterle F., Artusi A., Debattista K., Chalmers A. (2018). Advanced High Dynamic Range Imaging.

[6] Darmont A. (2019). High Dynamic Range Imaging: Sensors and Architectures.

[7] Dufaux F., Le Callet P., Mantiuk R., Mrak M. (2016). High Dynamic Range Video: From Acquisition, to Display and Applications.

[8] Reinhard E., Heidrich W., Debevec P., Pattanaik S., Ward G., Myszkowski K. (2010). High Dynamic Range Imaging: Acquisition, Display, and Image-Based Lighting.

[9] Parraga C.A., Otazu X. (2018). Which tone-mapping operator is the best? A comparative study of perceptual quality. JOSA A.

[10] Ward G. (1994). A contrast-based scalefactor for luminance display. Graph. Gems.

[11] Tumblin J., Rushmeier H. (1993). Tone reproduction for realistic images. IEEE Comput. Graph. Appl..

[12] Drago F., Myszkowski K., Annen T., Chiba N. (2003). Adaptive logarithmic mapping for displaying high contrast scenes. Comput. Graph. Forum.

[13] Reinhard E., Stark M., Shirley P., Ferwerda J. Photographic tone reproduction for digital images. Proceedings of the 29th Annual Conference on Computer Graphics and Interactive Techniques.

[14] Reinhard E., Devlin K. (2005). Dynamic range reduction inspired by photoreceptor physiology. IEEE Trans. Vis. Comput. Graph..

[15] Ferwerda J.A., Pattanaik S.N., Shirley P., Greenberg D.P. A model of visual adaptation for realistic image synthesis. Proceedings of the 23rd Annual Conference on Computer Graphics and Interactive Techniques.

[16] Ramsey S.D., Johnson J.T., Hansen C. Adaptive temporal tone mapping. In Proceedings of the 7th IASTED International Conference on Computer Graphics and Imaging.

[17] Van Hateren J.H. (2006). Encoding of high dynamic range video with a model of human cones. ACM Trans. Graph. (TOG).

[18] Irawan P., Ferwerda J.A., Marschner S.R. Perceptually Based Tone Mapping of High Dynamic Range Image Streams. Proceedings of the Eurographics Symposium on Rendering.

[19] Mantiuk R., Daly S., Kerofsky L. Display adaptive tone mapping. Proceedings of the SIGGRAPH ’08: Special Interest Group on Computer Graphics and Interactive Techniques Conference.

[20] Durand F., Dorsey J. Fast bilateral filtering for the display of high-dynamic-range images. Proceedings of the 29th Annual Conference on Computer Graphics and Interactive Techniques.

[21] Fattal R., Lischinski D., Werman M. Gradient domain high dynamic range compression. Proceedings of the 29th Annual Conference on Computer Graphics and Interactive Techniques.

[22] Kuang J., Johnson G.M., Fairchild M.D. (2007). iCAM06: A refined image appearance model for HDR image rendering. J. Vis. Commun. Image Represent..

[23] Meylan L., Alleysson D., Süsstrunk S. (2007). Model of retinal local adaptation for the tone mapping of color filter array images. JOSA A.

[24] Rahman Z.U., Jobson D.J., Woodell G.A. Multiscale retinex for color rendition and dynamic range compression. Proceedings of the Third IEEE International Conference on Image Processing (ICIP’96).

[25] Pattanaik S.N., Ferwerda J.A., Fairchild M.D., Greenberg D.P. A multiscale model of adaptation and spatial vision for realistic image display. Proceedings of the 25th Annual Conference on Computer Graphics and Interactive Techniques.

[26] Tumblin J., Turk G. LCIS: A boundary hierarchy for detail-preserving contrast reduction. Proceedings of the 26th Annual Conference on Computer Graphics and Interactive Techniques.

[27] Pattanaik S., Yee H. Adaptive gain control for high dynamic range image display. Proceedings of the 18th Spring Conference on Computer Graphics.

[28] Farbman Z., Fattal R., Lischinski D., Szeliski R. (2008). Edge-preserving decompositions for multi-scale tone and detail manipulation. ACM Trans. Graph. (TOG).

[29] Fattal R. (2009). Edge-avoiding wavelets and their applications. ACM Trans. Graph. (TOG).

[30] Larson G.W., Rushmeier H., Piatko C. (1997). A visibility matching tone reproduction operator for high dynamic range scenes. IEEE Trans. Vis. Comput. Graph..

[31] Eilertsen G., Mantiuk R.K., Unger J. (2015). Real-time noise-aware tone mapping. ACM Trans. Graph. (TOG).

[32] Rana A., Singh P., Valenzise G., Dufaux F., Komodakis N., Smolic A. (2019). Deep tone mapping operator for high dynamic range images. IEEE Trans. Image Process..

[33] Le C., Yan J., Fang Y., Ma K. (2021). Perceptually Optimized Deep High-Dynamic-Range Image Tone Mapping. arXiv.

[34] Vinker Y., Huberman-Spiegelglas I., Fattal R. Unpaired Learning for High Dynamic Range Image Tone Mapping. Proceedings of the IEEE/CVF International Conference on Computer Vision.

[35] Panetta K., Kezebou L., Oludare V., Agaian S., Xia Z. (2021). Tmo-net: A parameter-free tone mapping operator using generative adversarial network, and performance benchmarking on large scale hdr dataset. IEEE Access.

[36] Wang C., Chen B., Seidel H.P., Myszkowski K., Serrano A. (2022). Learning a self-supervised tone mapping operator via feature contrast masking loss. Comput. Graph. Forum.

[37] Zhang J., Wang Y., Tohidypour H., Pourazad M.T., Nasiopoulos P. A Generative Adversarial Network Based Tone Mapping Operator for 4K HDR Images. Proceedings of the 2023 International Conference on Computing, Networking and Communications (ICNC).

[38] Hu L., Chen H., Allebach J.P. Joint Multi-Scale Tone Mapping and Denoising for HDR Image Enhancement. Proceedings of the IEEE/CVF Winter Conference on Applications of Computer Vision.

[39] Mukherjee R., Melo M., Filipe V., Chalmers A., Bessa M. (2020). Backward compatible object detection using HDR image content. IEEE Access.

[40] Mukherjee R., Bessa M., Melo-Pinto P., Chalmers A. (2021). Object detection under challenging lighting conditions using high dynamic range imagery. IEEE Access.

[41] Onzon E., Mannan F., Heide F. Neural auto-exposure for high-dynamic range object detection. Proceedings of the IEEE/CVF Conference on Computer Vision and Pattern Recognition.

[42] Kocdemir I.H., Koz A., Akyuz A.O., Chalmers A., Alatan A., Kalkan S. (2023). Tmo-Det: Deep Tone-Mapping Optimized with and for Object Detection. Pattern Recognit. Lett..

[43] Bayer B.E. (1976). Color Imaging Array. U.S. Patent.

[44] Hirakawa K., Wolfe P.J. (2008). Spatio-Spectral Color Filter Array Design for Optimal Image Recovery. IEEE Trans. Image Process..

[45] Quad-Bayer Camera Sensors For Better Photos. https://www.ubergizmo.com/articles/quad-bayer-camera-sensor/.

[46] Sony Releases Stacked CMOS Image Sensor for Smartphones with Industry’s Highest 48 Effective Megapixels. https://www.sony.net/SonyInfo/News/Press/201807/18-060E/index.html.

[47] Lukac R., Plataniotis K. (2005). Color filter arrays: Design and performance analysis. IEEE Trans. Consum. Electron..

[48] Condat L. A new random color filter array with good spectral properties. Proceedings of the 2009 16th IEEE International Conference on Image Processing (ICIP).

[49] Gunturk B., Glotzbach J., Altunbasak Y., Schafer R., Mersereau R. (2005). Demosaicking: Color filter array interpolation. IEEE Signal Process. Mag..

[50] Li X., Gunturk B., Zhang L. Image demosaicing: A systematic survey. Proceedings of the Visual Communications and Image Processing 2008.

[51] Menon D., Calvagno G. (2011). Color image demosaicking: An overview. Signal Process. Image Commun..

[52] Wang Y., Cao R., Guan Y., Liu T., Yu Z. A deep survey in the Applications of demosaicking. Proceedings of the 2021 3rd International Academic Exchange Conference on Science and Technology Innovation (IAECST).

[53] Kwan C., Larkin J. (2019). Demosaicing of bayer and CFA 2.0 patterns for low lighting images. Electronics.

[54] Alleysson D., Susstrunk S., Herault J. (2005). Linear demosaicing inspired by the human visual system. IEEE Trans. Image Process..

[55] Aelterman J., Goossens B., De Vylder J., Pižurica A., Philips W. (2013). Computationally efficient locally adaptive demosaicing of color filter array images using the dual-tree complex wavelet packet transform. PLoS ONE.

[56] Menon D., Andriani S., Calvagno G. (2007). Demosaicing With Directional Filtering and a posteriori Decision. IEEE Trans. Image Process..

[57] Monno Y., Kiku D., Tanaka M., Okutomi M. (2017). Adaptive Residual Interpolation for Color and Multispectral Image Demosaicking. Sensors.

[58] Monno Y., Kikuchi S., Tanaka M., Okutomi M. (2015). A Practical One-Shot Multispectral Imaging System Using a Single Image Sensor. IEEE Trans. Image Process..

[59] Zhang C., Li Y., Wang J., Hao P. (2016). Universal Demosaicking of Color Filter Arrays. IEEE Trans. Image Process..

[60] Cui K., Jin Z., Steinbach E. Color Image Demosaicking Using a 3-Stage Convolutional Neural Network Structure. Proceedings of the 2018 25th IEEE International Conference on Image Processing (ICIP).

[61] Stojkovic A., Shopovska I., Luong H., Aelterman J., Jovanov L., Philips W. (2019). The Effect of the Color Filter Array Layout Choice on State-of-the-Art Demosaicing. Sensors.

[62] Park Y., Lee S., Jeong B., Yoon J. (2020). Joint demosaicing and denoising based on a variational deep image prior neural network. Sensors.

[63] Xu Y., Liu Z., Wu X., Chen W., Wen C., Li Z. (2021). Deep joint demosaicing and high dynamic range imaging within a single shot. IEEE Trans. Circuits Syst. Video Technol..

[64] Guo S., Liang Z., Zhang L. (2021). Joint denoising and demosaicking with green channel prior for real-world burst images. IEEE Trans. Image Process..

[65] Zhang T., Fu Y., Li C. Deep Spatial Adaptive Network for Real Image Demosaicing. Proceedings of the AAAI Conference on Artificial Intelligence.

[66] Gharbi M., Chaurasia G., Paris S., Durand F. (2016). Deep Joint Demosaicking and Denoising. ACM Trans. Graph..

[67] Kokkinos F., Lefkimmiatis S. (2018). Iterative Residual Network for Deep Joint Image Demosaicking and Denoising. arXiv.

[68] Marnerides D., Bashford-Rogers T., Hatchett J., Debattista K. (2018). ExpandNet: A Deep Convolutional Neural Network for High Dynamic Range Expansion from Low Dynamic Range Content. Comput. Graph. Forum.

[69] Git Repository for the ExpandNet Algorithm. https://github.com/dmarnerides/hdr-expandnet.

[70] Yu F., Chen H., Wang X., Xian W., Chen Y., Liu F., Madhavan V., Darrell T. Bdd100k: A diverse driving dataset for heterogeneous multitask learning. Proceedings of the IEEE/CVF Conference on Computer Vision and Pattern Recognition.

[71] BDD100K: A Large-scale Diverse Driving Video Database. https://bair.berkeley.edu/blog/2018/05/30/bdd/.

[72] Git Repository for the Implementation of “Berkeley Driving Dataset 100K. BDD100K: A Diverse Driving Dataset for Heterogeneous Multitask Learning”. https://github.com/bdd100k/bdd100k.

[73] Saruchi S. (2012). Adaptive sigmoid function to enhance low contrast images. Int. J. Comput. Appl..

[74] Helland T. (2012). Converting Temperature (Kelvin) to RGB: An Overview. https://tannerhelland.com/2012/09/18/convert-temperature-rgb-algorithm-code.html.

[75] Hirakawa K., Parks T.W. (2006). Joint demosaicing and denoising. IEEE Trans. Image Process..

[76] Liu L., Jia X., Liu J., Tian Q. Joint demosaicing and denoising with self guidance. Proceedings of the IEEE/CVF Conference on Computer Vision and Pattern Recognition.

[77] Git Repository for the “Unpaired Learning for High Dynamic Range Image Tone Mapping” Algorithm. https://github.com/yael-vinker/unpaired_hdr_tmo/.

[78] Arcos-Garcia A., Alvarez-Garcia J.A., Soria-Morillo L.M. (2018). Evaluation of deep neural networks for traffic sign detection systems. Neurocomputing.

[79] Git Repository for “Evaluation of Deep Neural Networks for Traffic Sign Detection Systems”. https://github.com/aarcosg/traffic-sign-detection.

